# Genome‐wide association study identifies 7q11.22 and 7q36.3 associated with noise‐induced hearing loss among Chinese population

**DOI:** 10.1111/jcmm.16094

**Published:** 2020-11-26

**Authors:** Yuguang Niu, Chengyong Xie, Zhenhua Du, Jifeng Zeng, Hongxia Chen, Liang Jin, Qing Zhang, Huiying Yu, Yahui Wang, Jie Ping, Chenning Yang, Xinyi Liu, Yuanfeng Li, Gangqiao Zhou

**Affiliations:** ^1^ Department of Otolaryngology the First Medical Center of PLA General Hospital Beijing China; ^2^ Medical College of Guizhou University Guiyang city China; ^3^ State Key Laboratory of Proteomics National Center for Protein Sciences Beijing Institute of Radiation Medicine Beijing China; ^4^ Department of Otolaryngology the No. 954 Hospital of PLA Shannan City China; ^5^ Collaborative Innovation Center for Personalized Cancer Medicine Center for Global Health School of Public Health Nanjing Medical University Nanjing City China; ^6^ Outpatient Department the Fifth Medical Center of PLA General Hospital Beijing China

**Keywords:** *AUTS2*, genome‐wide association study, noise‐induced hearing loss, *PTPRN2*, *WDR60*

## Abstract

Noise‐induced hearing loss (NIHL) seriously affects the life quality of humans and causes huge economic losses to society. To identify novel genetic loci involved in NIHL, we conducted a genome‐wide association study (GWAS) for this symptom in Chinese populations. GWAS scan was performed in 89 NIHL subjects (cases) and 209 subjects with normal hearing who have been exposed to a similar noise environment (controls), followed by a replication study consisting of 53 cases and 360 controls. We identified that four candidate pathways were nominally significantly associated with NIHL, including the Erbb, Wnt, hedgehog and intraflagellar transport pathways. In addition, two novel index single‐nucleotide polymorphisms, rs35075890 in the intron of *AUTS2* gene at 7q11.22 (combined *P* = 1.3 × 10^−6^) and rs10081191 in the intron of *PTPRN2* gene at 7q36.3 (combined *P* = 2.1 × 10^−6^), were significantly associated with NIHL. Furthermore, the expression quantitative trait loci analyses revealed that in brain tissues, the genotypes of rs35075890 are significantly associated with the expression levels of *AUTS2*, and the genotypes of rs10081191 are significantly associated with the expressions of *PTPRN2* and *WDR60*. In conclusion, our findings highlight two novel loci at 7q11.22 and 7q36.3 conferring susceptibility to NIHL.

## 1 INTRODUCTION

Noise is one of the most widespread environmental pollutions in living environments.^1^ Regular noise exposure may cause noise‐induced hearing loss (NIHL). As millions of people are daily exposed to harmful levels of noise, approximately 5% of the population worldwide suffers from industrial, military or recreational noise.^2^ Thus, NIHL seriously affects the life quality of humans and causes huge economic losses to society.^3^

Noise‐induced hearing loss was considered to be a multifactorial and polygenic event involved genetic and environmental components.^4^ The known environmental factors include noise, smoking, organic solvent exposure, higher blood pressure and cholesterol.^5^, ^6^ Animal studies have confirmed that genetic factors are involved in NIHL.^7^, ^8^, ^9^ Strains of mice that exhibit age‐related hearing loss (ARHL) were more susceptible to noise than other strains of mice.^8^, ^10^ Besides, knockout mice, such as *Pmca2* ^−/−^, *Sod1* ^−/−^, *Gpx1* ^−/−^ and *Cdh23* ^+/‐^, were more susceptible to noise than wild‐type mice in the same litter.^9^ Twin studies on humans have revealed that genetic components played important roles in the development of NIHL.^11^ Candidate gene‐based association studies have identified that several single‐nucleotide polymorphisms (SNPs) were significantly associated with the risk of NIHL. Genes tagged by these SNPs were mainly cataloged into the following four gene sets^9^: (a) oxidative stress genes, such as *CAT*, *SOD1* and *SOD2*; (b) inner ear potassium recycling pathway genes, such as *KCNQ4* and *KCNE1*; (c) *HSP* genes, such as *Hsp70*; and (d) monogenic deafness genes, such as *PCDH15* and *MYH14*. Recently, a genome‐wide association study (GWAS) in a European population consisting of 25 cases and 23 controls has revealed that one SNP (rs7598759) mapped in the nucleolin (*NCL*) gene was significantly correlated with the risk of NIHL.^12^ However, these findings are not enough to explain the total heritability of NIHL. In addition, the sample size of the existed GWAS for NIHL was limited, and GWAS for NIHL in Chinese population has never been performed.

To identify novel genes related to susceptibility to NIHL in the Chinese population, we here conducted a GWAS for NIHL in 89 NIHL patients (cases) and 209 subjects with normal hearing who have been exposed to the similar noise environment (controls), followed by a replication study in an independent sample set consisting of 53 NIHL cases and 360 controls. We found strong evidence for 7q11.22 (index rs35075890) and 7q36.3 (index rs10081191) as new loci contributing to susceptibility to NIHL. These findings expanded our understanding of the genetic basis of NIHL.

## 2 MATERIALS AND METHODS

### 2.1 Study participants

In the present study, we conducted a two‐stage GWAS among the Chinese populations, totally consisting of 711 Chinese males. The discovery stage contains 298 subjects and the replication stage contains 413 subjects (Table S1). All the subjects were recruited from occupational noise‐exposed workers from a single factory between March 2018 and September 2019 from Bengbu city at Anhui province. These workers were exposed to noise greater than 100 decibels (dB) for more than 8 hours (h) per day. All subjects had no hearing‐related complications, ear trauma, usage of certain drugs or toxins and otitis media. Pure tone audiometry was performed in an audiometric booth for each subject, by trained physicians using a Madsen Voyager 522 audiometer (Kastrup, Denmark) according to standard procedures. Pure tone audiometry was performed at least 12 h after the last exposure to noise in the workplace to avoid the temporary threshold shift.

The hearing level of the smallest sound of the subject's ears was detected at frequencies of 250 Hertz (Hz), 500 Hz, 1000 Hz, 2000 Hz, 4000 Hz and 8000 Hz, respectively. According to the classification of the World Health Organization, if the minimum threshold of hearing on either side of the ears of a subject is greater than 25 dB, this subject can be regarded as a case of NIHL.^13^ The other information, including demographic factors, noise exposure time, noise exposure intensity and hearing threshold of both ears after noise exposure, was also collected by a structured questionnaire. According to this criterion, the discovery stage contains 89 cases and 209 controls, and the replication stage contains 53 cases and 360 controls (Table S1).

#### 2.1.1 Discovery population

This population contained 298 individuals, consisting of 89 cases and 209 controls. All subjects were recruited from occupational noise‐exposed male workers in March, 2018 from Bengbu city at Anhui province, China. The mean age (SD) of the cases and controls is 23.8 (1.5) and 23.3 (1.6) years old, respectively (Table S1). All of them were genotyped using the Illumina Infinium Asian Screening Array‐24 (v1.0).

#### 2.1.2 Replication population

This population contained 413 individuals, consisting of 53 cases and 360 controls. All subjects were recruited from occupational noise‐exposed male workers between August 2018 and September 2019 from Bengbu city at Anhui province, China. The mean age (SD) of the cases and controls was 26.5 (5.7) and 24.5 (2.5) years old, respectively (Table S1).

Overall, in both the discovery stage and the replication stage, the mean age of the cases was significantly older than that of controls (*P* = .07 and 0.006, respectively; Table S1). In all genotyping experiments, the researchers were blind to the case/control status of all participants.

### 2.2 Genotyping and quality controls in the discovery stage

We performed strict quality control on both samples and SNPs to guarantee the subsequent robust association analyses.^14^, ^15^ Cases and controls were genotyped using the Illumina Infinium Asian Screening Array‐24 (v1.0), which consists of 659 184 SNPs. Briefly, samples were excluded if they (a) had an overall genotyping rate of <90%; (b) showed gender ambiguity; (c) showed unexpected duplicates or relatives (PI_HAT > 0.025), or (d) were identified as outliers. PCA was used to detect the outliers using Genome‐wide Complex Trait Analysis software (GCTA; version 1.92.2).^16^ SNPs were excluded if they had (a) a call rate of <90%; (b) a minor allele frequency (MAF) of <0.05; (c) did not map to autosomal chromosomes; and (d) *P*‐value is less than 1.0 × 10^−4^ in Hardy‐Weinberg equilibrium (HWE) test in discovery stage population. After quality controls, a total of 302 253 SNPs remained in all the 89 cases and 209 controls for subsequent analyses (Table S2).

### 2.3 Genome‐wide association analyses in the discovery stage

We used PLINK (version 1.90) to analyse the associations of genome‐wide SNPs with the risk of NIHL.^17^, ^18^ We carried out the logistic regression analysis under the additive model, adjusting for the age and noise exposure time. To adjust for population structure, we calculated the Tracy‐Widom (TW) statistic for the first 10 principal components (PCs) and found one significant PC (ie PC1) (*P* < .05). Thus, we carried out another logistic regression analysis under the additive model, adjusting for the age, noise exposure time and PC1. The Manhattan plot of ‐log10 (*P* values) was generated using R (version 3.5.0). The quantile‐quantile (Q‐Q) plot was generated using the R package to evaluate the overall significance of the genome‐wide associations and the potential impact of population stratification. A lambda (*λ*) expansion coefficient is given to indicate whether the system deviation exists.

### 2.4 Selection of SNPs for replication stage

A total of 262 SNPs with *P*‐value ≤ 1.0 × 10^−4^ in the discovery stage were selected for the replication study. These 262 SNPs were imported into Haploview (v4.2) software. By setting the linkage disequilibrium (LD) threshold (*r* ^2^ < 0.05), the genomic region where the SNPs showed strong LD were grouped as one locus. Thus, these 262 SNPs were grouped as a total of 29 loci. Then, the lead SNPs with the lowest *P*‐value in each locus were selected for the subsequent replication stage. Here, we gave priority to those genotyped SNPs rather than imputed ones, because even the lower imputation error rate may have a considerable impact on the downstream association analyses.^19^, ^20^

### 2.5 Statistical analyses

Meta‐analyses of the data generated from both the discovery stage and replication stage were conducted to assess the pooled genetic effects using the meta‐analysis helper (METAL) software. Cochran's *Q* statistic was calculated to test the heterogeneity between groups. Haploview software (version 4.2) was used to infer the LD structure of specific genomic regions. Regional plots were generated using the online tool LocusZoom1.1.^21^

### 2.6 Other analyses

Details of SNPs imputation, pathway‐based association analyses, genotyping/quality controls/association analyses in the replication stage, genotype‐expression association analyses, functional annotations of the candidate SNPs, colocalization analyses for GWAS and eQTL signals and power analyses are provided in the Supplementary materials and methods.

## 3 RESULTS

### 3.1 Genome‐wide association analyses

**Figure 1:**
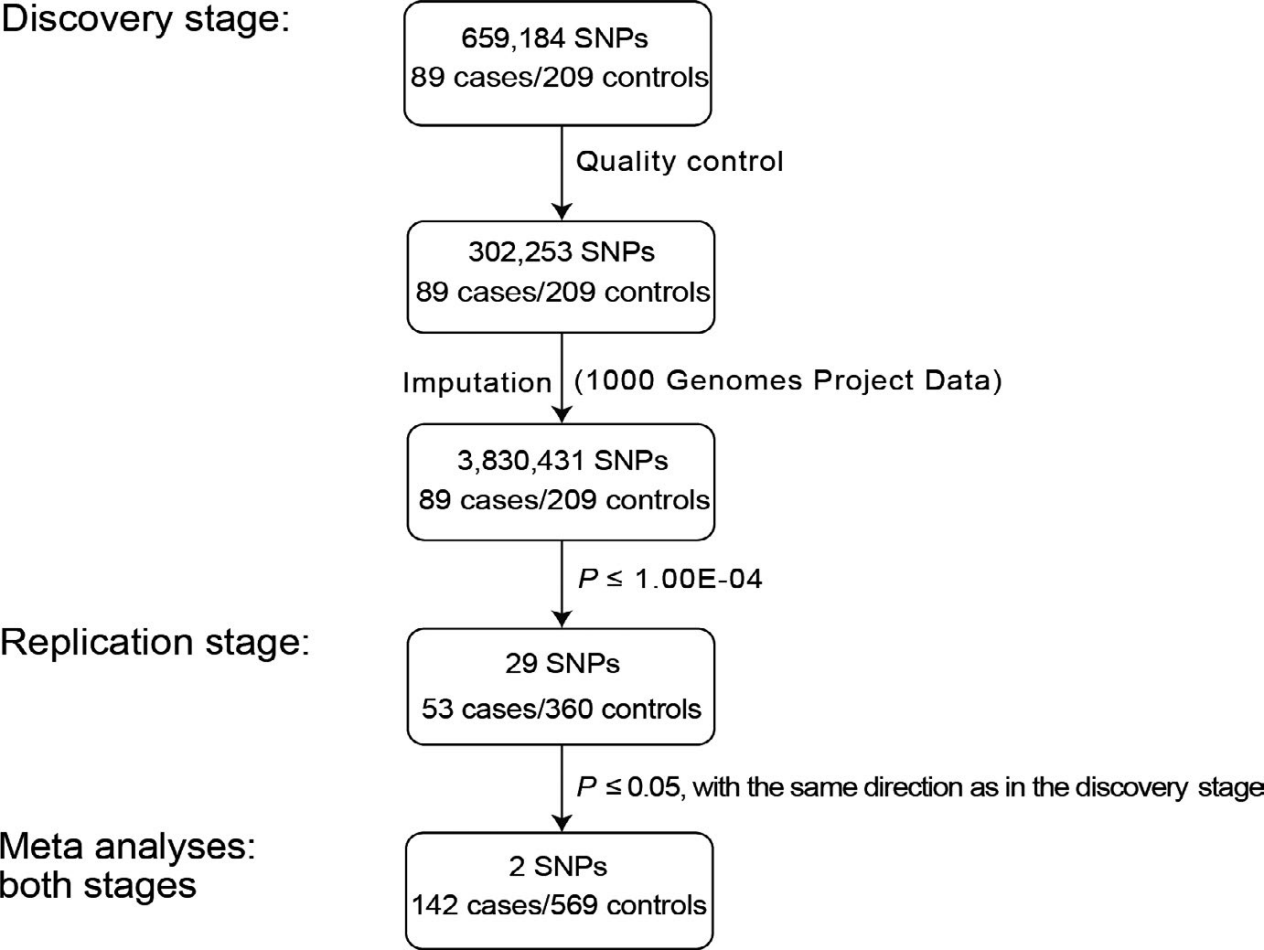
An overview of the study workflow. Numbers refer to the cases and controls and the SNPs genotyped or imputed. Imputation was performed using the data from the 1000 Genomes Project and generated genotypes of 3 830 431 SNPs in the discovery stage. The 29 top significantly associated SNPs in the discovery stage (*P* ≤ 1.0 × 10^−4^) were genotyped in the samples in the replication stage. Two index SNPs, rs35075890 and rs10081191, were replicated in the replication stage. Lastly, meta‐analyses combining two stages for rs35075890 and rs10081191 were performed

To detect novel loci conferring susceptibility to NIHL in the Chinese population, we carried out a two‐stage GWAS (Figure 1). In the discovery stage, a total of 298 Chinese male individuals, consisting of 89 cases and 209 controls (Table 1), were genotyped using the Illumina Infinium Asian Screening Array‐24 (v1.0). After quality controls, a total of 302 253 autosomal SNPs in 298 individuals survived, with an average genotyping call rate > 99.8% in this case‐control population (Table S2). No outlier was presented using the principal component analyses (PCA) (Figure S1A‐C). PCA also showed that all subjects were of Chinese ancestry (Figure S1D).

**Table 1:** Summary of the samples used in the discovery and replication stages

Stages	Cases		Controls	
	Sample size	Mean age (SD)	Sample size	Mean age (SD)
Discovery stage	89	23.8 (1.5)	209	23.3 (1.6)
Replication stage	53	26.5 (5.7)	360	24.5 (2.5)
Overall	142	24.8 (3.9)	569	24.1 (2.3)

Note

Pure tone audiometry was performed on each subject, and the hearing level of the smallest sound of the subject's ears was detected at the frequencies of 250 Hertz (Hz), 500 Hz, 1000 Hz, 2000 Hz, 4000 Hz and 8000 Hz, respectively. According to the classification of the World Health Organization, the cases were defined as those subjects whose minimum threshold of hearing on either side of the ears is greater than 25 decibels (dB), and the other subjects were defined as the controls.

Abbreviations: GWAS, genome‐wide association study; SD, standard deviation.

**Figure 2:**
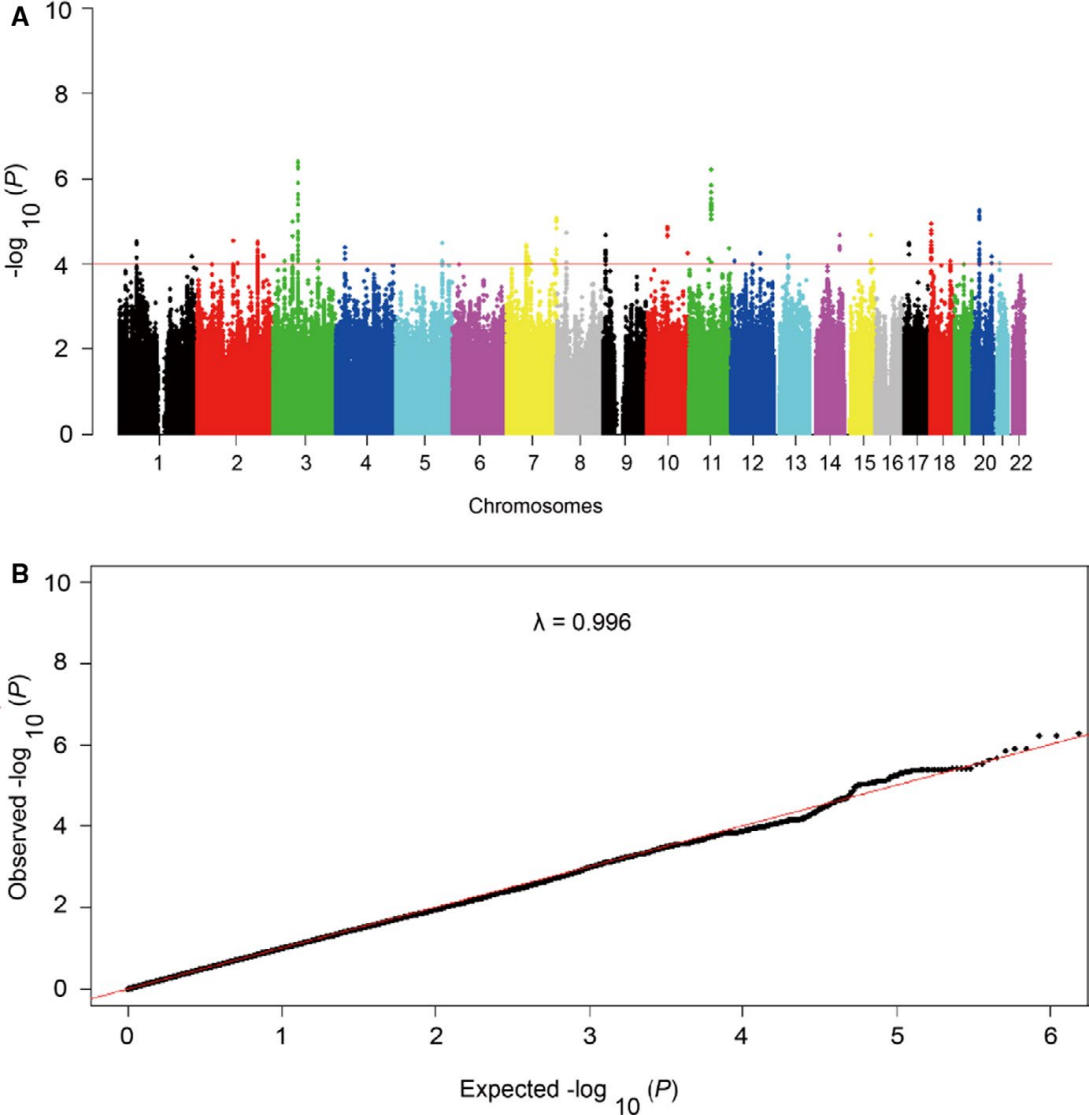
Manhattan plot and Quantile‐quantile plot of the genome‐wide *P* values in the discovery stage. A, The Manhattan plot of genome‐wide *P* values for the genotyped and imputed SNPs using logistic regression analyses in the discovery stage under the additive model. The *x*‐axis represents the genomic position (based on NCBI Build 37), and the *y*‐axis shows the −log_10_ (*P*). The red line represents the *P*‐value of 1.0 × 10^−4^. We selected candidate SNPs with *P*‐value ≤ 1.0 × 10^−4^ for replication. B, The quantile‐quantile plot. The red line represents the null hypothesis of no true association. The black line with gradient *λ* (inflation coefficient) is fitted to the lower 90% of the distribution of the observed test statistics. The plot is based on the genotyped and imputed SNPs that passed the quality controls. The value of the inflation factor is 0.996

To extend the coverage of the genomic region in the discovery stage, we used the SNPs genotypes that passed strict quality inspection to impute SNPs genotypes across the genome‐wide of all subjects. We used data from the 1000 Genomic Projects as a reference data set for our imputation and generated genotypes of 3 830 431 SNPs in 298 individuals (Table S2). Then, the genotype‐phenotype association analysis was performed by logistic regression model with the age and noise exposure time adjusted.^22^ We drew a plot of Manhattan to show the association between genome‐wide SNPs and the risk of NIHL (Figure 2A). The quantile‐quantile plot shows a good match between the observed *P*‐value distributions and the expected *P*‐value distribution by chance (*λ* = 0.996; Figure 2B), which indicates that the overall inflation of genome‐wide statistical results is minor at the discovery stage.

### 3.2 Several previously reported SNPs were replicated

Several candidate gene‐based association studies and GWASs have identified several SNPs relevant to the risk of NIHL.^9^ However, in the present study, these SNPs did not show significant associations (Table S3). These results were unlikely to be the result of imputation errors, as these SNPs were either directly genotyped or imputed with high quality. The inconsistent associations may be due to different ethnic populations or the limited number of samples.

We also reviewed the results from GWASs and the previous candidate gene‐based association studies on several other types of hearing loss. We observed that several SNPs in *SCARNA16*, *IQGAP2*, *PTPRK*, *GLI3*, *ADARB2* and *NDUFV2*, which have been reported to be significantly associated with several other types of hearing loss, were also significantly associated with NIHL in this study (all *P* values ≤ 0.05; Table S3). These results, therefore, indicated that NIHL and other types of hearing loss may share a common genetic mechanism, consistent with the hypothesis from previous studies.^23^

### 3.3 Pathway enrichment analyses

We conducted pathways enrichment analyses using Multi‐marker Analysis of GenoMic Annotation (MAGMA) software,^24^ based on the genome‐wide SNP *P* values. In these analyses, four candidate gene sets were identified to be nominally significantly associated with NIHL, including Erbb, Wnt, intraflagellar transport (IFT) and hedgehog pathways (all *P* < .05; Table S4). However, no gene set reached a significant threshold accounting for multiple testing correction (all false discovery rate [FDR]>0.05; Table S4). Further studies are needed to verify whether these four gene sets are associated with NIHL. Intriguingly, all of these four candidate gene sets were shown to be biological plausibility in the development of NIHL. For details, see Supplementary results.

### 3.4 Two new susceptibility loci at 7q11.22 AND 7q36.3 were identified

**Figure 3:**
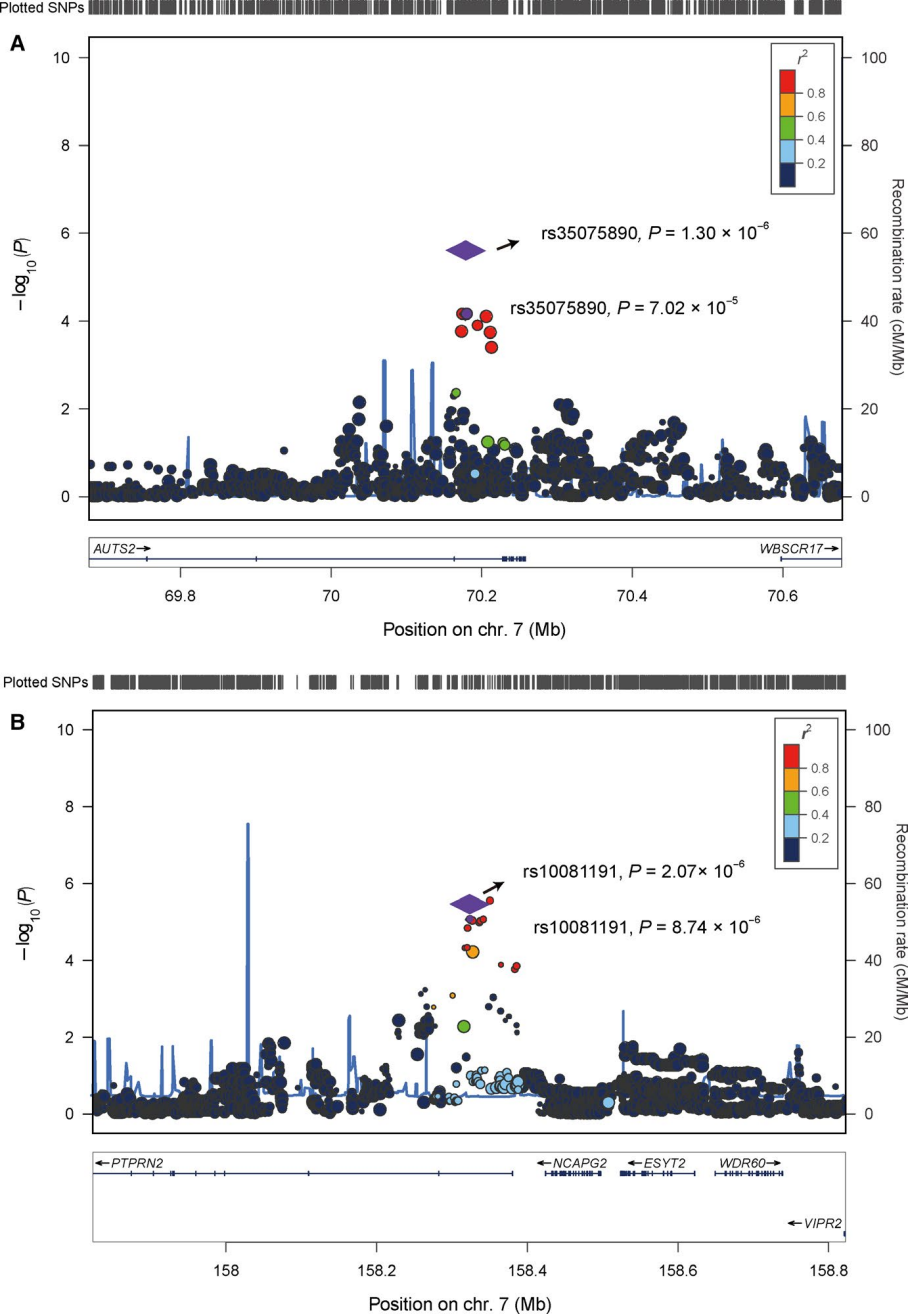
Regional plots for the associations in regions surrounding the rs35075890 and rs10081191 in the discovery stage. Genomic positions are based on NCBI Build 37. In the meta‐analyses, the *P* values of the SNPs were shown as purple diamonds, with their initial *P* values in the discovery stage shown as purple dots. The linkage disequilibrium (LD) values (*r* ^2^) to rs35075890 and rs10081191 for the other SNPs are indicated by marker colour. Red signifies *r* ^2^ > 0.8, orange 0.6 < *r* ^2^ ≤ 0.8, green 0.4 < *r* ^2^ ≤ 0.6, light blue 0.2 < *r* ^2^ ≤ 0.4 and blue *r* ^2^ ≤ 0.2. Estimated recombination rates, which are derived from the Asian population of the 1000 Genomes Project (Version 3, Nov 2014), are plotted in blue. Genes within the 500 Kb region surrounding the index SNPs rs35075890 (A) and rs10081191 **(B**) are annotated, with the positions of transcripts shown by arrows

In total, 29 SNPs showed significant associations with *P ≤ *1.0 × 10^−4^ in the discovery stage (Table S5). To adjust for population structure, we calculated the TW‐statistic for the top 10 PCs and found one significant PC (PC1) (*P* < .05). Thus, we carried out another logistic regression analysis under the additive model, adjusting for the age, noise exposure time and PC1, for the top 29 SNPs. The effect sizes of these 29 SNPs in analysis adjusting PC1 were similar to the effect sizes in the primary analysis (Table S5). We then selected these top 29 signals for replication in an independent sample set (Tables S5and S6). The inclusion and exclusion criteria were the same as those in the discovery stage, which consisting of 53 cases and 360 controls. All subjects were also Chinese male individuals (Table S1). Among these 29 SNPs, two ones were consistent with the same direction as observed in the discovery stage (for index rs35075890 at 7q11.22, *P* = .0035, OR = 2.21 and for rs10081191 at 7q36.3, *P* = .020, OR = 1.52; Table S7). We then combined the results of the discovery stage and the replication stage for these two SNPs. Both two SNPs showed a more significant associations (OR = 3.17, *P* = 1.3 × 10^−6^ for rs35075890; and OR = 1.99, *P* = 2.1 × 10^−6^ for rs10081191; Table 2, Figure 3, Table S7 and Figure S2). No evidence of heterogeneity for OR values of rs35075890 and rs10081191 was observed among all the sample sets (*P*_heterogeneity_ = 0.34 and 0.11, respectively; Table 2).

**Table 2:** Association results for the index rs35075890 and rs10081191 in the case/control populations

SNPs	Chr. (Cytoband)	Studies	Cases^a^	Controls^a^	ORs (95% CIs)	*P* values	*I* ^2^	*P*_heterogeneity_
rs35075890	7q11.22	Discovery stage	1/20/67	0/13/193	4.53 (2.15‐9.54)	7.02 × 10^−5^	0.22	.34
G/A^b^		Replication stage	0/12/39	1/40/313	2.21 (1.09‐4.47)	3.47 × 10^−3^		
		Overall	1/32/106	1/53/506	3.17 (1.91‐4.55)	1.30 × 10^−6^		
rs10081191	7q36.3	Discovery stage	16/45/24	10/78/105	2.58 (1.70‐3.92)	8.74 × 10^−6^	0.47	.11
A/C^b^		Replication stage	12/19/22	37/142/178	1.52 (1.02‐2.27)	2.03 × 10^−2^		
		Overall	28/64/46	47/220/283	1.99 (1.51‐2.65)	2.07 × 10^−6^		

Abbreviations: Chr., chromosome. CI, confidence interval; OR, odds ratio; SNP, single‐nucleotide polymorphism.

^a^ Counts of the GG/GA/AA genotypes for rs35075890, and the AA/AC/CC genotypes for rs10081191 in the case/control populations, respectively. The number of genotyped samples varies due to genotyping failure.

^b^ Minor allele/major allele. ORs and 95% CIs were calculated under the additive model by logistic regression analyses while adjusting for the age and noise exposure time. By using the meta‐analysis helper (METAL) software, we calculated the heterogeneity of the population in the discovery stage and the replication stage. *P*_heterogeneity_ value of less than 0.05 was considered to be statistically significant.

The influence of rs35075890 and rs10081191 on NIHL was further investigated by stratification for age. In the pooled case‐control sample, we found no significant changes in the effects of rs35075890 and rs10081191 by age‐stratified subgroups (*P*_heterogeneity_ = 0.99 and 0.57, respectively; Table S8). In addition, the rs35075890 was directly genotyped with a high call rate (99%), and the rs10081191 was imputed with high imputation quality (imputation info score 0.98), suggesting the associations between these two loci and NIHL were unlikely to be false positives caused by the errors of genotyping or imputation. The interaction effects between rs35075890, rs10081191 and noise exposure time were not assessed, because of the lack of noise exposure time in several samples (n = 19) in the replication stage. Therefore, the possibility that the association signals detected by rs3507890 and rs10081191 can be attributed to the risk of noise exposure time to NIHL cannot be completely excluded.

### 3.5 *AUTS2* was identified as the candidate gene at 7q11.22

The SNP rs35075890 is located at the sixth intron of *AUTS2* gene at chromosome 7q11.22. Two genes (*AUTS2* and *WBSCR17*) are located within 1 megabase (Mb) of genomic regions surrounding this SNP (Figure 3A). To identify the candidate gene tagged by rs35075890 at 7q11.22, we performed the expression quantitative trait loci (eQTL) analyses based on the expression profile data of brain tissues from the Genotype‐Tissue Expression (GTEx) (v8). We focused on brain tissues, as previous neuroscience studies have identified neural changes related to tinnitus that commence at the cochlear nucleus and extend to the auditory cortex and other brain regions.^25^ The eQTL analyses showed that the protective allele A of rs35075890 was significantly correlated with the lower expression levels of *AUST2* in the frontal cortex, hippocampus and hypothalamus (*P = *2.9 × 10^−3^, 7.5 × 10^−3^ and 2.0 × 10^−2^, respectively; Figure S3 and Table S9A), but not with the expression levels of *WBSCR17*. These associations were also significant even after Bonferroni correction for multiple comparisons (n = 2). Colocalization analysis using LocusCompare web tool (http://locuscompare.com/) further showed that the NIHL‐associated SNP rs35075890 was colocalized with eQTL signals for *AUTS2* in brain tissues (Figure S4). *AUTS2* has been shown to affect the development of the nervous system,^26^ including the eighth cranial nerve, which is closely related to auditory conduction.^27^ Accordingly, *AUTS2* was involved in many types of neurological diseases, including autism spectrum disorders (ASD), intellectual disability and developmental delay.^28^ However, AUTS2 has not been reported previously to be relevant to NIHL. The potential roles for AUTS2 in the development of NIHL are warranted in the future.

It is worth noting that in the initial GWAS analysis, the candidate causal SNP is not necessarily the most statistically significant.^29^ Thus, to investigate the candidate causative variants at 7q11.22, we annotated the genetic variants that are tagged by the index SNP rs35075890 (*r* ^2^ > 0.4). Using the publicly available software HaploReg (v4.1), we observed that all the SNPs in strong LD with rs35075890 are located at *AUTS2* intron regions (Table S10A). Further, by using the Probabilistic Annotation Integrator (PAINTOR) software, we get the posterior probability of six SNPs tagged by the index SNP rs35075890. Among them, the SNP rs79299033 achieved the highest posterior probability in this locus (*P*_posterior_ = 0.69; Table S10A), suggesting that rs79299033 may be the candidate causative SNP at 7q11.22. On the basis of the data of multiple human brain tissues from the Encyclopedia of DNA Elements (ENCODE), we revealed that rs79299033 is located at an enhancer region, where is also a DNase I hypersensitive site (DHS) in human foetal brain tissue (Table S10A). These observations indicated that rs79299033 was the most likely variant having a causative effect on NIHL.

### 3.6 *PTPRN2* and *WDR60* were identified as the candidate genes at 7q36.3

The SNP rs10081191 is located at the first intron of *PTPRN2* at chromosome 7q36.3. Five protein‐coding genes (including *PTPRN2*, *NCAPG2*, *ESYT2*, *WDR60* and *VIPR2*) are located within 1 Mb region surrounding the rs10081191 (Figure 3B). We performed eQTL analyses on the basis of the data from brain tissues in GTEx to identify the potentially causative gene(s) at 7q36.3. The eQTL analyses showed that the protective allele C of rs10081191 is significantly related to the higher expression levels of *PTPRN2* and *WDR60* in brain tissues (based on Bonferroni correction, *P* < .05/5 were considered to be statistically significant; Table S9B and Figures S5 and S6). Colocalization analysis using LocusCompare web tool (http://locuscompare.com/) further showed that the NIHL‐associated SNP rs10081191 is colocalized with eQTL signals for *PTPRN2*, but not for *WDR60*, in brain tissues (Figure S4). *PTPRN2* encodes a member of the protein tyrosine phosphatases (PTP) family. This protein has been identified as an autoantigen in insulin‐dependent diabetes mellitus.^30^ Interestingly, the *PTPRN2* gene mutation has been shown to cause bilateral Duane retraction syndrome with severe bilateral hearing loss.^31^ *WDR60* gene encodes a member of the WD‐repeat protein family. *WDR60* is required for IFT and plays an important role in the formation of cilia.^32^ The dysfunction of cilia can cause multiple system diseases including hearing loss.^33^ It is worth noting that IFT is one of the significantly associated gene sets in this study (Table S4). Collectively, these results suggested a potential role for the *WDR60* gene and IFT gene set in NIHL. Further studies are needed to confirm the role of *PTPRN2* or *WDR60* in the development of NIHL.

To identify the potential causative variants at 7q36.3 locus, we annotated the genetic variants tagged by the index SNP rs10081191 (*r* ^2^ > 0.4) by using HaploReg. All the SNPs in strong LD with rs10081191 are located at *PTPRN2* intron regions except rs4909237, which is a variant at a non‐coding transcript *MIR595*. By using the PAINTOR software, we get the posterior probability of 14 SNPs at 7q36.3 locus. There were two SNPs, rs4909237 and rs10270958, with posterior probabilities higher than 0.90 (Table S10B). We observed that the rs4909237 is located at enhancers in multiple types of brain tissues. In addition, this SNP is located at the DHSs of foetal brain tissue (Table S10B). However, rs10270958 was not located in genomic regulatory regions in brain tissues (Table S10B). These results indicated that the candidate causative variants at 7q36.3 may regulate the expression of the nearby genes (*PTPRN2* or *WDR60*) in brain tissues.

## 4 DISCUSSION

In the present study, we conducted a GWAS on the risk of NIHL. We identified two novel loci at 7q11.22 (index rs35075890) and 7q36.3 (index rs10081191) contributing to the genetic susceptibility to NIHL. To our best knowledge, this is the first GWAS for genetic susceptibility to NIHL in the Chinese population.

To gain insight into the racial frequency distributions of the index rs35075890 and rs10081191 in different populations, we compared the allele frequencies of these two SNPs in this study with that in the four main populations from the 1000 Genomes Project. We found that the rs35075890[G] allele frequency in this study (0.064) was similar to that in East Asian descent (0.078, *P* = .34), but significantly less than that in Europeans (0.313, *P* = 1.7 × 10^−38^), Africans (0.154, *P* = 1.7 × 10^−9^) and Americans (0.210, *P* = 2.8 × 10^−16^) of the 1000 Genomes Project (Table S11). Meanwhile, the rs10081191[A] allele frequency in this study (0.315) was similar to that in East Asian descent of the 1000 Genomes Project (0.315, *P* = .99), but also significantly less than those in Europeans (0.494, *P* = 1.5 × 10^−14^), Africans (0.381, *P* = 2.0 × 10^−3^ and Americans (0.465, *P* = 2.5 × 10^−9^) of the 1000 Genomes Project (Table S11). Whether these differences among different races will affect the susceptibility of NIHL remains to be determined.

The rs35075890 and rs10081191 are located in the non‐coding regions at 7q11.22 and 7q36.3, respectively. As non‐coding SNPs may alter gene expression levels, we used eQTL analyses to explore whether the rs35075890, rs10081191 or SNPs tagged by them are cis‐acting regulators of the nearby gene(s) in human brain tissues. Indeed, we found that the results of the eQTL analyses concerning *AUTS2*, *PTPRN2* and *WDR60* were statistically significant in multiple types of brain tissues. However, it remains possible that the genes other than *AUTS2*, *PTPRN2* and *WDR60* are involved in NIHL within these two loci. Additionally, because of the variety of genome copy number, genome methylation and gene expression, tissue heterogeneity makes eQTL analysis more complicated. Therefore, caution needs to be applied in the analyses and interpretation of eQTL analysis. Additional analyses in larger sample sizes and more cell or tissue types relevant to the aetiology of NIHL will be needed to confirm the biological plausibility of *AUTS2*, *PTPRN2* and *WDR60* as candidate susceptibility genes for NIHL.

*AUTS2* encodes a nuclear protein, which may be involved in transcriptional regulation.^34^ *AUTS2* is expressed in different organisms, and mainly in the brain tissues.^34^ Further analysis of the expression of Auts2 in mouse brain revealed that the gene is expressed in the developing cerebral cortex and cerebellum.^35^ *AUTS2* is associated with multiple mental disorders, including ASD, intellectual disability and schizophrenia.^36^ No evidence has ever shown that *AUTS2* was involved in NIHL. However, it was proposed that there is a lot of overlapping symptoms in ASD and hearing loss, and these two kinds of diseases tended to occur simultaneously.^37^ According to the Third National Health and Nutrition Examination Survey of the Center for Disease Control and Prevention from 1988 to 1994, the incidence of at least one abnormal auditory function in children with ASDs was higher than that in the control group or the general population.^38^ Thus, the finding in this study that *AUTS2* is a potential NIHL susceptibility gene provided genetic evidence for this phenomenon.

*PTPRN2* encodes a member of the PTP family. PTPs remove phosphate groups from tyrosine residues, and thereby influence signal transduction and then influence cellular processes such as metabolism, motility and survival.^39^ As for *PTPRN2*, it has been reported to be involved in the regulation of insulin secretion in response to glucose stimuli.^40^ Previous studies have shown that *PTPRN2* was a biologically relevant candidate gene for metabolic diseases, such as obesity, diabetes and cancers.^40^, ^41^ Recently, *PTPRN2* genomic duplication has been shown to be linked to severe bilateral hearing loss.^31^ In addition to *PTPRN2*, several other PTP family members were also reported to be related to hearing loss. For example, *PTP1B* inhibition was reported to be helpful in the treatment of deafness associated with hyperglycaemia and Type II diabetes.^42^ Mutation of *PTPRD* was found to be related to hearing loss, growth retardation and intellectual disability.^43^ *PTPRQ* mutations were identified to be related to congenital hearing loss and vestibular dysfunction syndrome.^44^ Interestingly, a previous GWAS identified that *PTPRK* (index rs10499138) is significantly associated with hearing loss.^45^ We replicated this genetic association in NIHL in this study (*P* = .03; Table S3). Taken together, these results suggested that *PTPRN2* is a plausible susceptibility gene for NIHL. Recently, an increasing number of PTPs is being proposed as clinically relevant targets.^46^ Thus, our findings highlighted the relevance of *PTPRN2* as a novel therapeutic target for NIHL by PTP inhibitors.

*WDR60*, which contains four WD repeat sequences, encodes a member of the WD‐repeat protein family.^32^ Several members of this family are involved in a variety of cellular processes, including cell cycle progression, signal transduction, apoptosis and gene regulation.^47^ WDR60 and another family member WDR34 form dynein‐2 motor complex, which moves other proteins and cell materials within cilia, and plays crucial roles in primary cilia formation and function.^32^ Consistently, in human telomerase reverse transcriptase‐immortalized retinal pigment epithelial 1 (hTERT‐RPE1) cells, which have been used in several studies analysing ciliary assembly and function, knockout of *WDR60* showed defects in retrograde ciliary protein trafficking.^47^ The cilia on the inner and outer hair cells can convert mechanical deflection signals into electrochemical signals, and participate in the transmission of the sound.^48^ Dysfunctions in cilium assembly can lead to ciliopathies, systemic diseases including hearing loss, olfactory loss and retinal dystrophy.^33^ Collectively, these observations suggested that *WDR60* is a candidate gene for the genetic susceptibility to NIHL.

In this study, the prevalence of NIHL increased in individuals who carry the at‐risk rs35075890 [G] and rs10081191[A] allele. With more susceptibility loci of NIHL being identified in future studies, polygenic risk scores based on these loci together with rs35075890 and rs10081191 will help to define the high‐risk populations of NIHL among noise exposure workers, and finally improve NIHL prevention.

**Figure 4:**
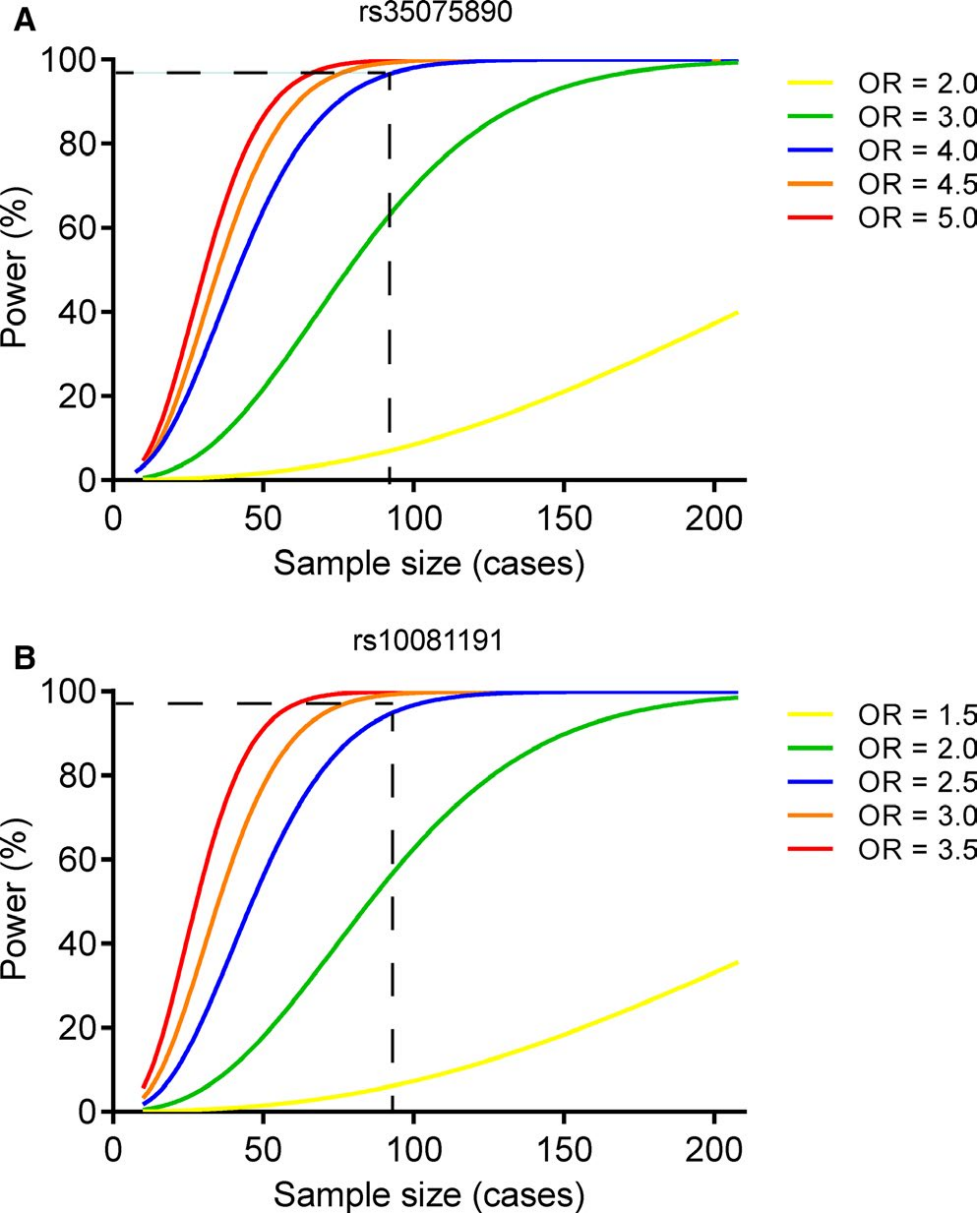
Power to detect the genetic effects of rs35075890 and rs10081191. Vertical and horizontal dashed lines show the power of rs35075890 (A) and rs10081191 (B), giving the noise‐induced hearing loss prevalence being 5%, their minor allele frequencies being 0.06 and 0.315, in 89 cases and 209 controls, respectively. OR, odds ratio

The advantage of the present study is that the selection of the case‐control population is strict. The cases and controls were exposed to a similar noise environment. All the cases and controls were males, and the ages of the cases and controls are similar, excluding the confounding interference of the gender and age factors. The shortcoming of this study is that the sample size remains limited. However, our GWAS reveals enough statistical power in 89 cases and 209 controls to detect the index rs35075890 (OR = 4.53; MAF = 0.06) and rs10081191 (OR = 2.58; MAF = 0.32), with estimated powers to be ~98.92% and ~98.89%, respectively (Figure 4). We admit that our GWAS has limited power to detect SNPs which had moderate effects. Further replications were needed.

## 5 CONCLUSION

Our findings highlighted two novel loci (7q11.22 and 7q36.3) conferring genetic susceptibility to NIHL in Chinese populations.

## CONFLICT OF INTEREST

The authors confirm that there are no conflicts of interest.

## AUTHOR CONTRIBUTION

**Yuguang Niu:** Conceptualization (equal). **Chengyong Xie:** Conceptualization (supporting); Data curation (supporting). **Zhenhua Du:** Data curation (supporting). **Jifeng Zeng:** Project administration (supporting). **Hongxia Chen:** Funding acquisition (supporting). **Liang Jin:** Formal analysis (supporting). **Qing Zhang:** Data curation (supporting). **Huiying Yu:** Data curation (supporting). **Yahui Wang:** Data curation (supporting). **Jie Ping:** Writing‐review & editing (supporting). **Chenning Yang:** Writing‐review & editing (supporting). **Xinyi Liu:** Writing‐original draft (supporting); Writing‐review & editing (supporting). **Yuanfeng Li:** Conceptualization (equal); Data curation (lead); Funding acquisition (equal); Supervision (equal); Validation (equal). **Gangqiao Zhou:** Conceptualization (lead); Data curation (lead); Supervision (lead); Validation (lead).

## ETHICAL APPROVAL

This study was performed with the approval of the Medical Ethical Committee of Beijing Institute of Radiation Medicine (Beijing, China). Written informed consent was obtained from each participant.

## Supporting information

Supplementary Material

## Data Availability

Data from this study are publicly available through the following link: http://cbportal.org/pubfiles/JCMM-07-2020-200.zip.
