# Supplementary material for: Genome‐wide association study identifies 7q11.22 and 7q36.3 associated with noise‐induced hearing loss among Chinese population
Source: J Cell Mol Med. 2020 Nov 26;25(1):411–20. doi: 10.1111/jcmm.16094 (PMC7810922; doi:10.1111/jcmm.16094)
Supplement: Supplementary file 1 — Supplementary Material [file JCMM-25-411-s001.doc]

**Supplementary Materials**

**Genome-wide association study identifies 7q11.22 and 7q36.3 associated with noise-induced hearing loss among Chinese population**

**Running** **title: Genome-wide association study of noise-induced hearing loss**

Yuguang Niu^1,#^, Chengyong Xie^2,#^, Zhenhua Du^3,#^, Jifeng Zeng^4^, Hongxia Chen^3^, Liang Jin^3^, Qing Zhang^5^, Huiying Yu^6^, Yahui Wang^3^, Jie Ping^3^, Chenning Yang^3^, Xinyi Liu^3^, Yuanfeng Li^3,*^, Gangqiao Zhou^2,3,5,*^

^1^Department of Otolaryngology, the First Medical Center of PLA General Hospital, Beijing, China;

^2^Medical College of Guizhou University, Guiyang city, China;

^3^State Key Laboratory of Proteomics, National Center for Protein Sciences, Beijing Institute of Radiation Medicine, Beijing, China;

^4^Department of Otolaryngology, the No.954 Hospital of PLA, Shannan city, China;

^5^Collaborative Innovation Center for Personalized Cancer Medicine, Center for Global Health, School of Public Health, Nanjing Medical University, Nanjing city, China;

^6^Outpatient Department, the Fifth Medical Center of PLA General Hospital, Beijing, China.

^#^These authors contributed equally to this work.

^*^Correspondence should be addressed to:

Dr. Gangqiao Zhou, State Key Laboratory of Proteomics, National Center for Protein Sciences, Beijing Institute of Radiation Medicine, 27 Taiping Road, Beijing, 100850, China. E-mail: zhougq114@126.com; Phone: 86-10-66931201.

OR

Dr. Yuanfeng Li, State Key Laboratory of Proteomics, National Center for Protein Sciences, Beijing Institute of Radiation Medicine, 27 Taiping Road, Beijing, 100850, China. E-mail: liyf_snp@163.com; Phone: 86-10-61777099.

**Index**

**Supplementary materials and methods**

**Supplementary results**

**Supplementary Figures:**

**Supplementary Figure 1** Principal components analyses (PCA) of samples in the discovery stage and reference samples from the 1000 Genomes Project data.

**Supplementary Figure 2** Forest plots for the index rs35075890 and rs10081191 across the discovery and replication stages.

**Supplementary Figure 3** The genotypes of rs35075890 are significantly associated with the expression levels of *AUTS2* in several types of brain tissues from GTEx.

**Supplementary Figure 4** Colocalization of association signals from GWAS analysis and eQTL analysis at 7q11.22 and 7q36.3.

**Supplementary Figure 5** The genotypes of rs10081191 are significantly associated with the expression levels of *PTPRN2* in several types of brain tissues from GTEx.

**Supplementary Figure 6** The genotypes of rs10081191 are significantly associated with the expression levels of *WDR60* in several types of brain tissues from GTEx.

**Supplementary Tables:**

**Supplementary Table 1** Summary of the case/control populations used in this study.

**Supplementary Table 2** Summary of the SNPs passing the quality controls.

**Supplementary Table 3** Summary of the SNPs that have been reported to be significantly associated with hearing loss in previous GWASs and candidate gene-based association studies.

**Supplementary Table 4** Pathways enrichment analyses in the discovery stage.

**Supplementary Table 5** Summary of the 29 top significantly associated SNPs in the discovery stage.

**Supplementary Table 6** Primers used for SNPs genotyping in the replication stage.

**Supplementary Table 7** Summary of the genetic association results for the 29 SNPs in the replication stage.

**Supplementary Table 8** Stratification analyses of rs35075890 and rs10081191 by age.

**Supplementary Table 9** The eQTL analyses of rs35075890 and rs10081191 in 13 types of human brain tissues from GTEx.

**Supplementary Table 10** The predicted functional relevance of rs35075890, rs10081191 and the other SNPs in strong LD with them.

**Supplementary Table 11** The allele and genotype frequencies of rs35075890 and rs10081191 in different populations.

**Supplementary materials and methods**

**SNPs imputation**

To increase the coverage of SNPs in the data sets and generate more genotypes in the discovery stage, we performed imputation on the GWAS genotyping data sets using the SHAPEIT software (version 2) and IMPUTE2 software (version 2.2.2). The 1,000 Genomes Project data (version 3, Mar 2012) was used as the reference dataset. The phased haplotypes constructed by SHAPEIT were directly inputted into IMPUTE2. A posterior probability of 0.60 was used as the threshold for calling genotypes. We then converted the imputed probabilities into hard genotype calls. Quality controls were then performed on these imputed SNPs. We retained the imputed SNPs which had (i) a call rate of > 90%; (ii) a MAF of > 0.05; or (iii) a *P*-value of greater than 1.0 × 10^-4^ in a Hardy-Weinberg equilibrium test. The multi-allelic SNPs (tetra-allelic SNPs and tri-allelic SNPs) were filtered out. Finally, we obtained a total of 3,830,413 SNPs in those 89 cases and 209 controls (**Supplementary Table 2**).

**Pathway‑based association analyses**

Multi-marker Analysis of GenoMic Annotation (MAGMA) [1] was used to perform the pathway-based associations in the genome-wide analysis in the discovery stage. Pathway-based association analysis is a new and effective strategy that can detect the associations missed by traditional GWAS and explore the biological mechanisms of diseases [1]. MAGMA used an F-test to compute the gene *P* values, and the gene *P* values were then used for the gene-set analysis with a regression model. A total of 186 gene sets from the Kyoto Encyclopedia of Genes and Genomes (KEGG) database, [289 gene sets](http://software.broadinstitute.org/gsea/msigdb/genesets.jsp?collection=CP:BIOCARTA) from the BioCarta database, [196 gene sets](http://software.broadinstitute.org/gsea/msigdb/genesets.jsp?collection=CP:PID) from the Pathway Interaction Database (PID), and [1,499 gene sets](http://software.broadinstitute.org/gsea/msigdb/genesets.jsp?collection=CP:REACTOME) from the Reactome database were included in our analyses. We performed multiple testing correction using the Benjamini-Hochberg (BH) method, and the false discovery rate (FDR) values of less than 0.05 were considered to be statistically significant.

**Genotyping, quality controls and association analyses in the replication stage**

We selected candidate SNPs for the replication stage. The candidate SNPs were defined as SNPs with *P*-value ≤ 1.0 × 10^-4^ in the discovery stage. Totally, we selected 29 candidate SNPs [2]. We genotyped these SNPs in the replication stage using Sequenom genotyping. Briefly, the locus-specific polymerase chain reaction and primers were designed using the MassARRAY Assay Design 3.0 software (Sequenom, Inc. USA). Approximately 15 ng of the genomic DNA for each sample was used to genotype these SNPs. The DNA samples were amplified by multiplex PCR, and the products were then used for locus-specific single-base extension reactions. The resulting products were desalted and transferred to a 384-element SpectroCHIP array (Sequenom). Allele detection was performed using MALDI-TOF-MS (Sequenom). The mass spectrograms were analyzed by the MassARRAY TYPER software. The cluster patterns of the genotyping data from the Sequenom assays were visually checked to confirm their good quality. The genotype data in the replication stage was subjected to the same quality control analyses as in the discovery stage. Among the 29 SNPs, rs56328361 was failed to be genotyped. Of the other 28 successfully genotyped SNPs, the mass spectrograms of these SNPs were analyzed by the MassARRAY TYPER software (Sequenom Inc., USA), and visually checked to confirm they're good quality. In the replication study, 5% of the individuals were randomly selected for repeated genotyping, and the consistent rate of the genotyping results was 100%. The genotype data in the replication studies were subjected to the same quality control as in the discovery stage. Only rs35075890 and rs10081191 were survived in the replication stage, with *P* < 0.05 and the same effect direction as the discovery stage. We performed the logistic regression analysis under the additive model using PLINK (version 1.90), adjusting for the age and noise exposure time. Because the noise exposure time of 19 participants was missing in the replication stage, we therefore replaced the missing data with the average noise exposure time of all participants in the replication stage (2.4 years). Thus, we could perform association analyses adjusting for noise exposure time in the replication stage.

**Genotype-expression association analyses**

We evaluated the genotype-specific expression of rs35075890 and rs10081191 in 13 types of human brain tissues *via* cis-expression quantitative trait loci (cis-eQTL) analysis, through the Genotype-Tissue Expression (GTEx, release v8) portal. We only focused on the protein-coding genes located within the 1 Mb region surrounding the SNPs. Violin plots of the genotype-specific expression were constructed to visualize the normalized gene expressions among three genotypes of rs35075890 (AA, AG and GG; G and A alleles indicate the minor and major allele types, respectively) and rs10081191 (AA, AC and CC; A and C alleles indicate the minor and major allele types, respectively). We performed Bonferroni correction to account for multiple testing for eQTL analyses involving multiple SNP-gene pairs, resulting in a significance threshold of *P* < 0.025 for 7q11.22 locus (*i.e.*, 0.05/2) and *P* < 0.01 for 7q36.3 locus (*i.e.*, 0.05/5), respectively.

**Functional annotations of the candidate SNPs at 7q11.22 and 7q36.3 loci**

Functional annotations of 6 SNPs that are tagged by rs35075890 at 7q11.22 and 14 SNPs that are tagged by rs10081191 at 7q36.3 ( *r*^2^ > 0.4) were performed using multiple tools, including the HaploReg and Probabilistic Annotation Integrator (PAINTOR) [3]. The PAINTOR method is a probabilistic framework, which incorporates several types of information including the association Z-scores from the summary statistics, functional annotations and LD matrix of pairwise correlation coefficients. The method integrates the association strength of genotype and phenotype with two independent sources of information, LD structure and functional annotation data to calculate the posterior probability to be a causal variant for each SNP across all fine-mapping loci. Before running the software, we set the following parameters: enumerate was set to 3, and four DNA elements (coding, promoter, enhancer and DNase I hypersensitive site [DHS]) were involved in the calculation of posteriori probabilities in the function annotation file.

**Colocalization analyses for GWAS and eQTL signals**

To identify colocalization events between the GWAS and eQTL signals, and to further suggest the candidate casual gene at 7q11.22 and 7q36.3, we performed colocalization analyses using LocusCompare web tool (<http://locuscompare.com/>). The eQTL data for rs35075890-*AUTS2* in brain frontal cortex tissues, rs10081191-*PTPRN2* in brain cortex tissues and rs10081191-*WDR60* in brain amygdala tissues were download from the GTEx (v8). We used eQTL data in these tissues, because the most significant eQTL signals for rs35075890-*AUTS2*, rs10081191-*PTPRN2* and rs10081191-*WDR60* were detected in these tissues, respectively. The GWAS data for rs35075890 and rs10081191 were extracted from this study. We then used these eQTL data and GWAS data to perform colocalization analyses. For 7q11.22 locus, the rs35075890 genotypes were significantly associated with the expression levels of *AUTS2*. The LocusCompare plot further indicated that the rs35075890 and *AUTS2* eQTL associations probably represent a true colocalization event. The lead eQTL variant (rs11764444, *P* = 2.1 × 10^-4^) showed a high linkage disequilibrium (LD) with rs35075890 (*r*^2^ = 0.9), and also had a significant GWAS *P* value (*P* = 1.3 × 10^-4^). For 7q36.3 locus, the index SNP rs10081191 genotypes were significantly associated with the expression levels of *PTPRN2* and *WDR60*. The LocusCompare plot at this region further indicated that the eQTL associations between rs10081191 and *PTPRN2* probably represent a true colocalization event, because the lead eQTL variant for *PTPRN2* (rs67224972, *P* = 2.6 × 10^-5^) showed a high LD (*r*^2^ = 0.9) with rs10081191, and had a significant GWAS *P* value (*P* = 1.8 × 10^-4^); while the lead eQTL variant for *WDR60* (rs6966681, *P* = 2.6 × 10^-5^) showed a low LD (*r*^2^ < 0.2) with rs10081191, and had a non-significant GWAS *P* value (*P* = 0.20).

**Power analyses**

Power analyses were performed using the Power for Genetic Association Analyses (PGA v1.1), which is a package of algorithms and graphical user interfaces developed in Matlab for power and sample size calculation under various genetic models and statistical constraints. Giving noise-induced hearing loss prevalence of 5%, 89 cases and 209 controls, and *P* value of 1.0 × 10^-4^, the power of our GWAS to identify the rs35075890 at 7q11.22 (OR = 4.53; MAF = 0.06) is ~98.92%, and the power to rs10081191 at 7q36.3 (OR = 2.58; MAF = 0.32) is ~98.89%.

**Supplementary results**

**Significantly associated pathways**

We conducted pathways enrichment analyses using Multi-marker Analysis of GenoMic Annotation (MAGMA) software [1]. In these analyses, four candidate gene sets were identified to be nominally significantly associated with NIHL with *P* < 0.05, including Erbb, Wnt, intraflagellar transport (IFT) and hedgehog pathways (**Supplementary Table 4**). However, no gene set reached a significant threshold accounting for multiple testing correction (all false discovery rate [FDR] < 0.05; **Supplementary Table 4**). Intriguingly, all of these four candidate gene sets may be involved in NIHL. For example, Erbb signaling is necessary for the long-term survival of adult spiral ganglion neurons in the inner ear [4], and the degeneration of spiral ganglion neurons could lead to hearing loss [5]. Wnt pathway is critical for cochlear development, converging on several key molecules to regulate prosensory specification, proliferation, hair cell differentiation, and cellular organization [6]. IFT is a complex bidirectional motility system that was required for the assembly and maintenance of cilia [7], and the mutations in IFT genes have been shown to cause several ciliopathies, which are characterized by hearing loss [8]. Hedgehog signaling plays an important role in hearing loss, promoting the proliferation of progenitor cells and subsequent hair cell formation in the cochlea of neonatal mice [9].

**Supplementary Figures:**


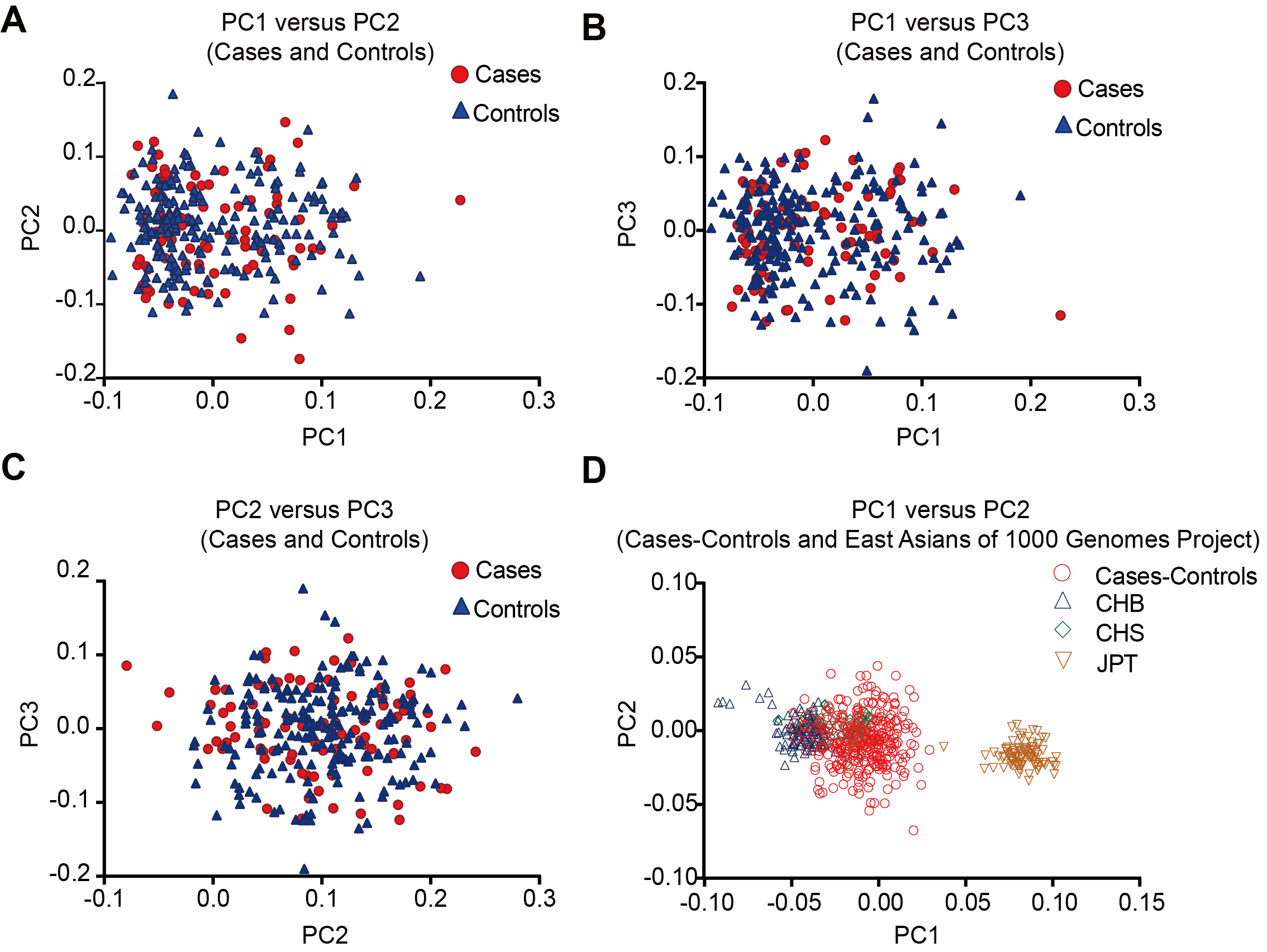


**Supplementary Figure** **1 Principal components analyses (PCA) of samples in the discovery stage and reference samples from the 1,000 Genomes Project data.**

(**A**) Principal component (PC) 1 *versus* PC2 for the cases (n = 89) and controls (n = 209) in the discovery stage of genome-wide association study (GWAS). (**B**) PC1 *versus* PC3 for the cases and controls in the discovery stage. (**C**) PC2 *versus* PC3 for the cases and controls in the discovery stage. Red represents the cases, and blue represents the controls. (**D**) PC1 *versus* PC2 for the cases and controls in the discovery stage (red), and for the reference individuals in the 1,000 Genomes Project data (n = 282), including 93 CHBs (Han Chinese in Beijing, China; blue), 100 CHSs (Southern Han Chinese, China; green) and 89 JPTs (Japanese in Tokyo, Japan; brown).


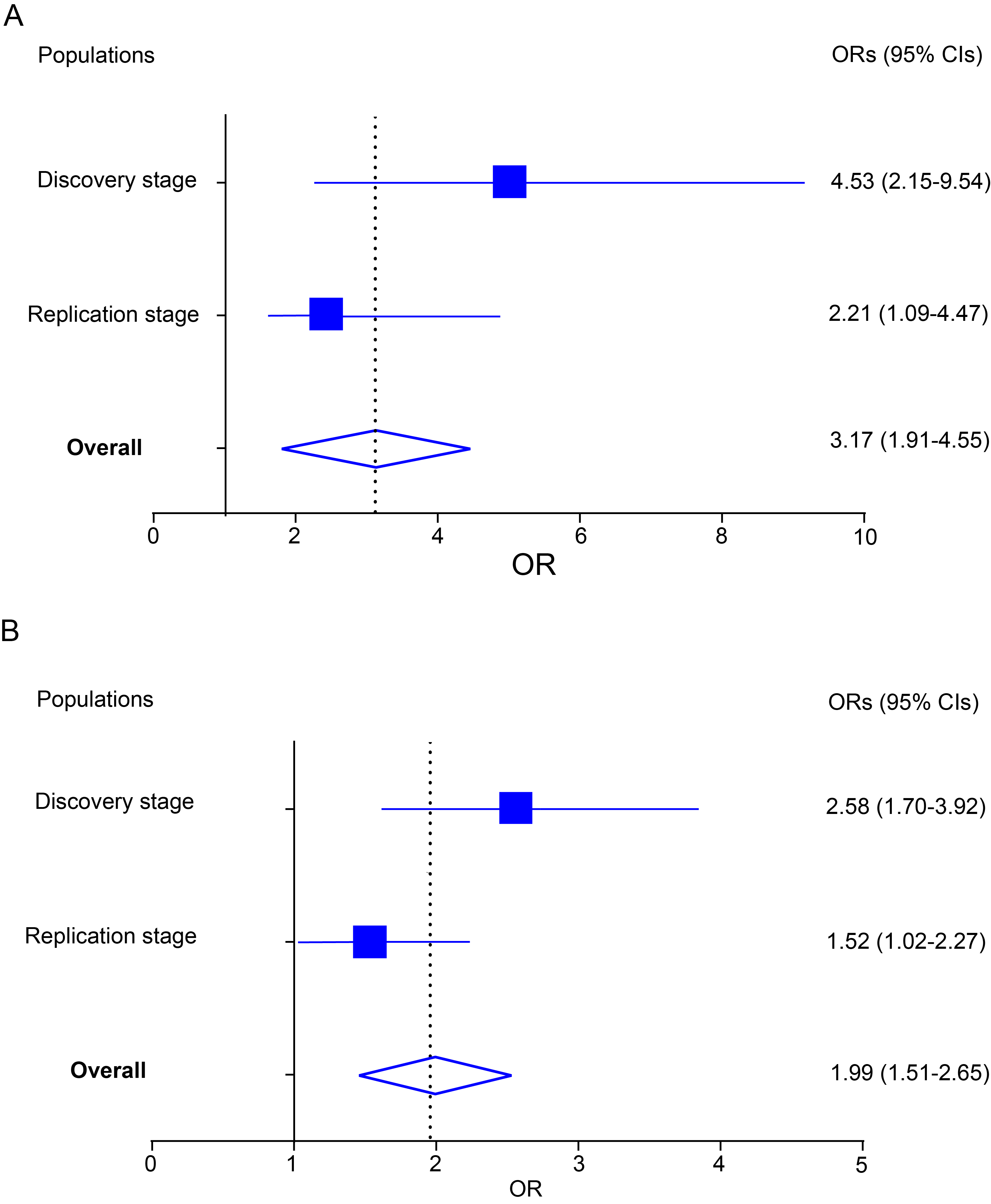


**Supplementary Figure 2 Forest plots for the index rs35075890 and rs10081191 across the discovery and replication stages.**

We plot the odds ratios (ORs; blue square) and the 95% confidence intervals (CIs; horizontal blue line) for each study. The two bars represent the results from two stages and the blue diamond below them summarizes their meta-analyses effects. The meta-analyses gave a joint *P* value of 1.3 × 10^-6^ (joint OR = 3.17, 95% CI = 1.91-4.55) for rs35075890 and 2.1 × 10^-6^ (OR = 1.99, 95% CI = 1.51-2.65) for rs10081191, respectively. A vertical black dashed line indicates the final OR of rs35075890 (**A**) or rs10081191 (**B**) by combining two stages.


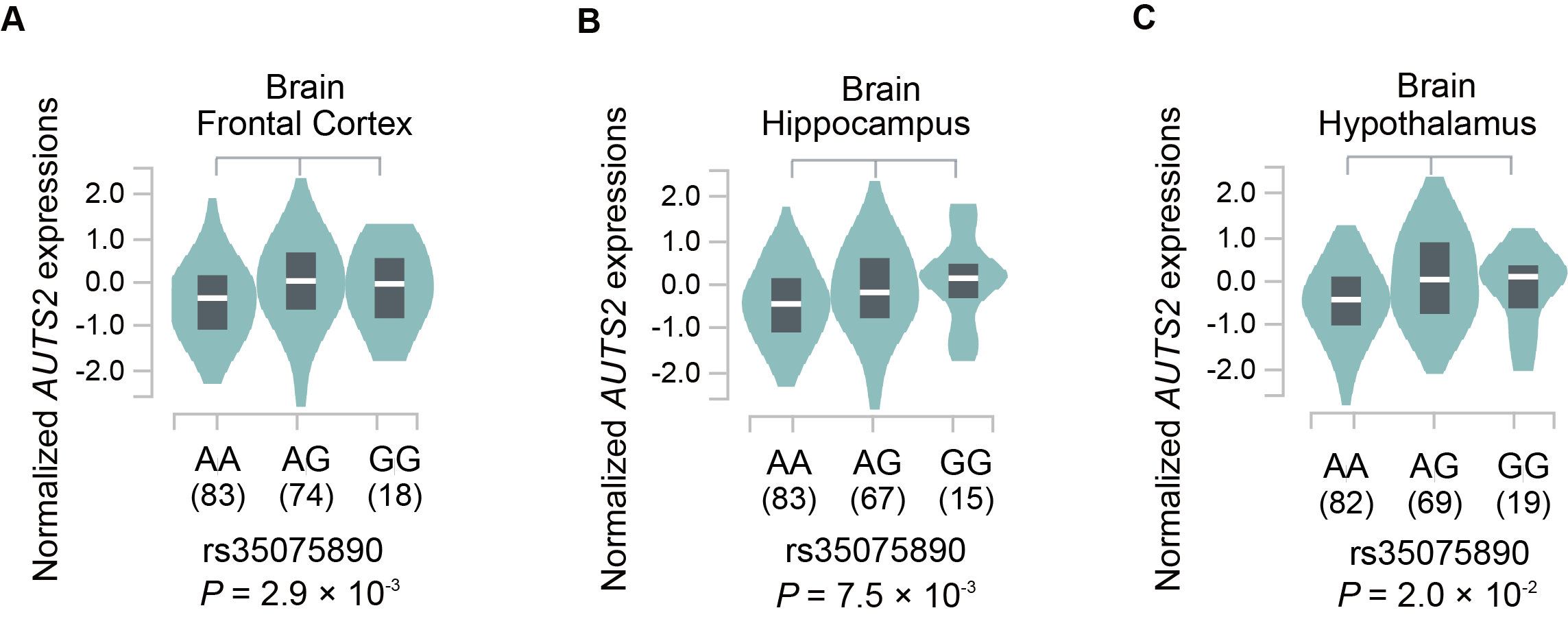


**Supplementary Figure 3 The genotypes of rs35075890 are significantly associated with the expression levels of *AUTS2* in several types of brain tissues from GTEx.**

The expression levels of *AUTS2* according to different rs35075890 genotypes (AA, AG and GG) in the frontal cortex (**A**), hippocampus (**B**) and hypothalamus (**C**) tissues of the brain were shown. The data of *AUTS2* mRNA expression levels were derived from GTEx. The *AUTS2* mRNA expression levels were normalized by the trimmed mean of M-values (TMM) approach and were then log2 transformed. The number below the genotype indicates the number of samples in each genotype. *P* value was generated using a linear regression model between the genotypes and expression levels. We also performed Bonferroni correction to account for multiple testing for eQTL analyses involving multiple SNP-gene pairs (n = 2), resulting in a significance threshold of *P* < 0.025 for 7q11.22. After multiple testing correction, the associations between *AUTS2* expression levels and rs35075890 genotypes (AA, AG and GG) remain significant in three brain regions (frontal cortex, hippocampus and hypothalamus). The white line in the box plot (black) shows the median value of the expression levels of each genotype. The eQTL associations with *P* < 0.05 are shown.


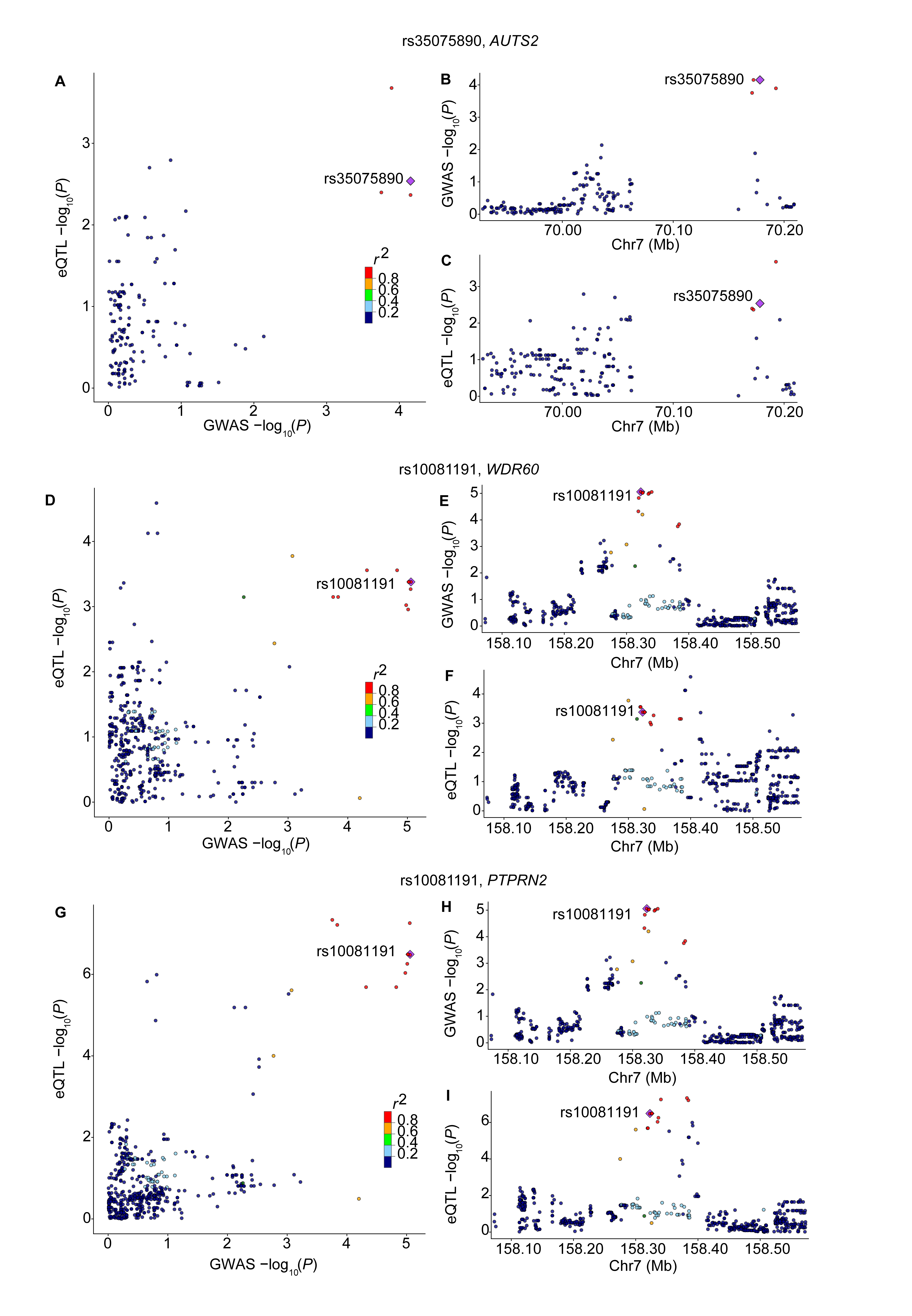


**Supplementary Figure 4 Colocalization of association signals from GWAS analysis and eQTL analysis at 7q11.22 and 7q36.3.**

LocusCompare plots for *AUTS2* (**A-C**), *WDR60* (**D-F**) and *PTPRN2* (**G-I**) at 7q11.22 and 7q36.3 were shown. Genomic positions are based on NCBI Build 37. For 7q11.22 locus, the rs35075890 genotypes were significantly associated with the expression levels of *AUTS2*. The LocusCompare plot further indicated that the rs35075890 and *AUTS2* eQTL associations probably represent a true colocalization event. The lead eQTL variant (rs11764444, *P* = 2.1 × 10^-4^) showed a high linkage disequilibrium (LD) with rs35075890 (*r*^2^ = 0.9), and also had a significant GWAS *P* value (*P* = 1.3 × 10^-4^). For 7q36.3 locus, the index SNP rs10081191 genotypes were significantly associated with the expression levels of *PTPRN2* and *WDR60*. The LocusCompare plot at this region further indicated that the eQTL associations between rs10081191 and *PTPRN2* probably represent a true colocalization event, because the lead eQTL variant for *PTPRN2* (rs67224972, *P* = 2.6 × 10^-5^) showed a high LD (*r*^2^ = 0.9) with rs10081191, and had a significant GWAS *P* value (*P* = 1.8 × 10^-4^); while the lead eQTL variant for *WDR60* (rs6966681, *P* = 2.6 × 10^-5^) showed a low LD (*r*^2^ < 0.2) with rs10081191, and had a non-significant GWAS *P* value (*P* = 0.20). The index SNPs rs35075890 and rs10081191 were shown as purple diamonds. The LD values (*r*^2^) to rs35075890 and rs10081191 for the other SNPs are indicated by marker color. Red signifies *r*^2^ > 0.8, orange 0.6 < *r*^2^ ≤ 0.8, green 0.4 < *r*^2^ ≤ 0.6, light blue 0.2 < *r*^2^ ≤ 0.4 and blue *r*^2^ ≤ 0.2.


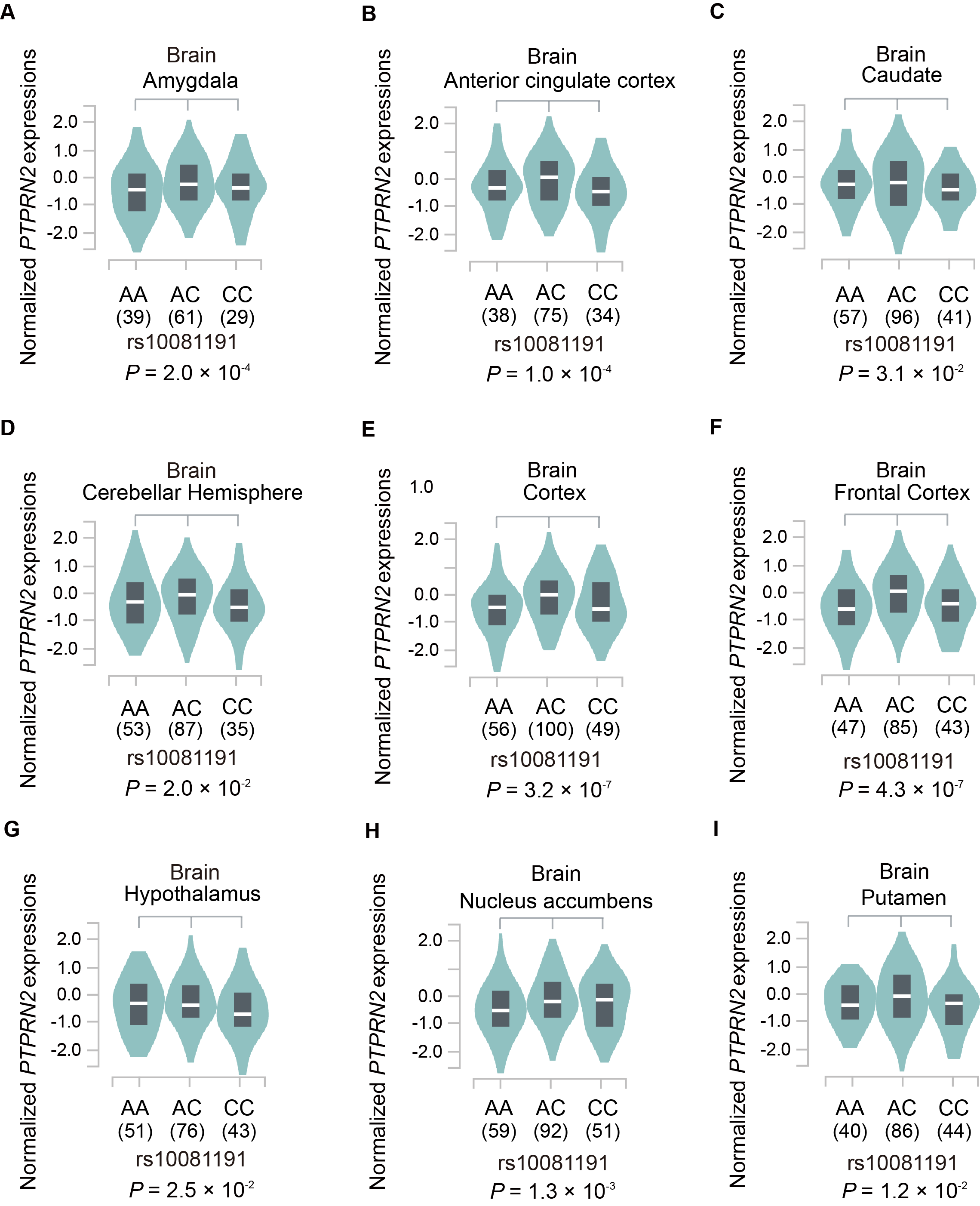


**Supplementary Figure 5 The genotypes of rs10081191 are significantly associated with the expression levels of *PTPRN2* in several types of brain tissues from GTEx.**

The expression levels of *PTPRN2* in different rs10081191 genotypes (AA, AC and CC) in the brain amygdala (**A**), anterior cingulate cortex (**B**), caudate (**C**), cerebellar hemisphere (**D**), cortex (**E**), frontal cortex (**F**), hypothalamus (**G**), nucleus accumbens (**H**) and putamen (**I**) were shown. The data of *AUTS2* mRNA expression levels were derived from GTEx. The *PTPRN2* mRNA expression levels were normalized by the trimmed mean of M-values (TMM) approach and were then log2 transformed. The number below the genotype indicates the number of samples in each genotype. *P* value was generated using a linear regression model between the genotypes and expression levels. We performed Bonferroni correction to account for multiple testing for eQTL analyses involving multiple SNP-gene pairs (n = 5), resulting in a significance threshold of *P* < 0.01 for 7q36.3. After multiple testing correction, the associations between *PTPRN2* expression levels and rs10081191 genotypes (AA, AC and CC) remain significant in tissues of five brain regions (amygdala, anterior cingulate cortex, cortex, frontal cortex and nucleus accumbens). The white line in the box plot (black) shows the median value of the expression of each genotype. The eQTL associations with *P* < 0.05 are shown.


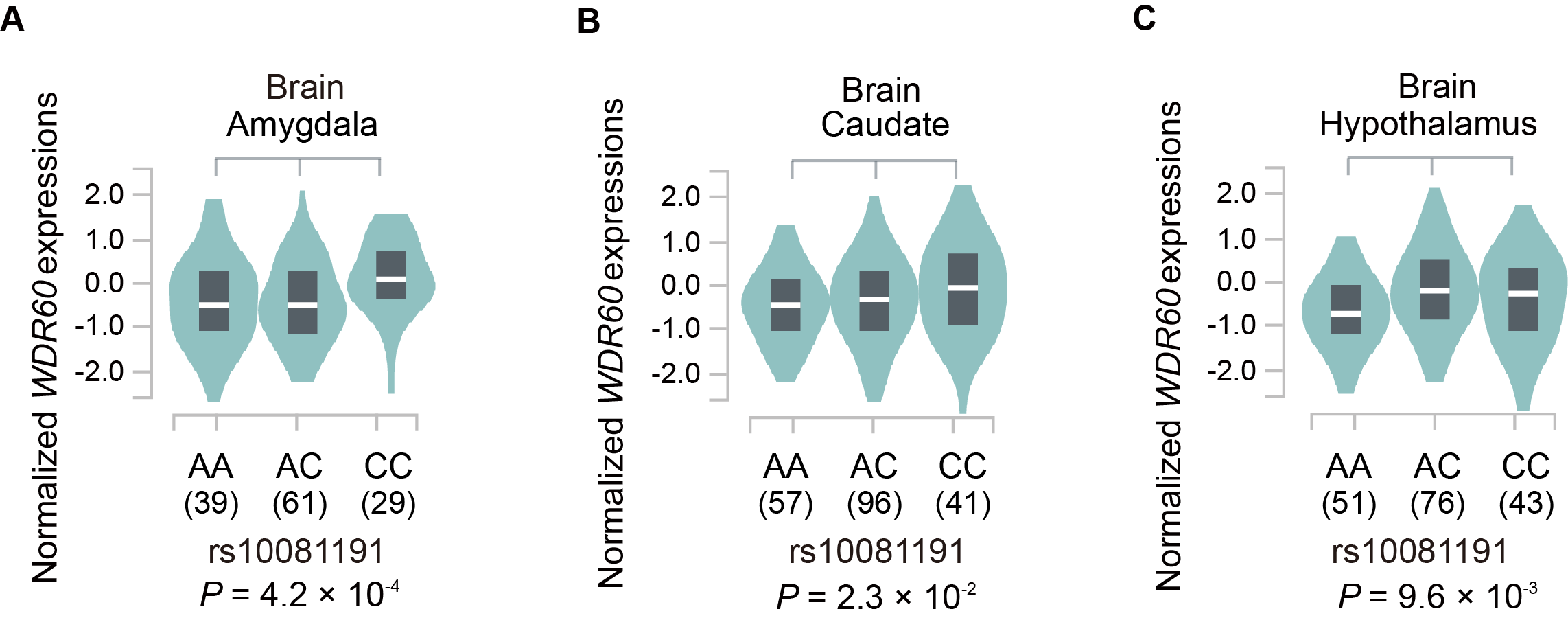


**Supplementary Figure 6 The genotypes of rs10081191 are significantly associated with the expression levels of *WDR60* in several types of brain tissues from GTEx.**

The expression levels of *WDR60* in different rs10081191 genotypes (AA, AC and CC) in the amygdala (**A**), caudate (**B**) and hypothalamus (**C**) were shown. The data of *AUTS2* mRNA expression levels were derived from GTEx. The *WDR60* mRNA expression levels were normalized by the trimmed mean of M-values (TMM) approach and were then log2 transformed. The number below the genotype indicates the number of samples in each genotype. *P* value was generated using a linear regression model between the genotypes and expression levels. We also performed Bonferroni correction to account for multiple testing for eQTL analyses involving multiple SNP-gene pairs (n = 5), resulting in a significance threshold of *P* < 0.01 for 7q36.3, after multiple testing correction, the associations between *WDR60* expression levels and rs10081191 genotypes (AA, AC and CC) remain significant in tissues of two brain regions (amygdala and hypothalamus). The white line in the box plot (black) shows the median value of the expression of each genotype. The eQTL associations with *P* < 0.05 are shown.

**Supplementary Table 1** Summary of the case/control populations used in this study.

| Categories | Discovery stage | | *P* | Replication stage | | *P* |
| --- | --- | --- | --- | --- | --- | --- |
|  | Cases | Controls |  | Cases | Controls |  |
| Age, years |  |  |  |  |  |  |
| Mean (SD) | 23.8 (1.5) | 23.3 (1.6) | 0.01 | 26.5 (5.7) | 24.5 (2.5) | 2.14E-05 |
| ≤ 23, n (%) | 34 (38.3) | 104 (49.8) | 0.07 | 10 (18.9) | 138 (38.3) | 0.006 |
| > 23, n (%) | 55 (61.7) | 105 (50.2) |  | 43 (81.1) | 222 (61.7) |  |
| Gender, n (%) |  |  |  |  |  | - |
| Male | 89 (100) | 209 (100) |  | 53 (100) | 360 (100) |  |
| Female | 0 (0) | 0 (0) |  | 0 (0) | 0 (0) |  |
| SD, standard deviation. -, not available. | | | | | | |

**Supplementary Table 2** Summary of the SNPs passed the quality controls.

| Chr. | Number of  genotyped  SNPs | Number of SNPs  with posterior probabilities > 0.6 | Number of imputed SNPs passed QC |
| --- | --- | --- | --- |
| 1 | 24,868 | 6,500,406 | 309,001 |
| 2 | 24,730 | 7,117,653 | 328,153 |
| 3 | 21,516 | 5,862,644 | 289,315 |
| 4 | 20,116 | 5,763,688 | 292,591 |
| 5 | 18,091 | 5,293,987 | 244,850 |
| 6 | 23,424 | 5,051,726 | 289,036 |
| 7 | 17,225 | 4,741,633 | 240,298 |
| 8 | 10,893 | 4,622,584 | 152,737 |
| 9 | 8,290 | 3,576,648 | 103,796 |
| 10 | 16,465 | 4,013,474 | 212,790 |
| 11 | 15,392 | 4,067,172 | 207,977 |
| 12 | 14,730 | 3,889,071 | 192,519 |
| 13 | 10,872 | 2,872,976 | 143,587 |
| 14 | 10,136 | 2,650,950 | 128,413 |
| 15 | 10,083 | 2,437,496 | 110,005 |
| 16 | 10,442 | 2,713,904 | 111,854 |
| 17 | 9,716 | 2,341,798 | 97,318 |
| 18 | 9,413 | 2,279,220 | 109,551 |
| 19 | 7,989 | 1,843,244 | 84,304 |
| 20 | 7,999 | 1,822,234 | 79,851 |
| 21 | 4,965 | 1,104,664 | 54,386 |
| 22 | 4,898 | 1,110,227 | 48,099 |
| Sum | 302,253 | 81,677,399 | 3,830,431. |
| Chr., chromosome; QC, quality control; SNP, single-nucleotide polymorphism; Imputation on the GWAS data sets was performed using the IMPUTE2 software (version 2.3.1), on the basis of the 1,000 Genomes Project data (version 3). | | | |
|

**Supplementary Table 3** Summary of the SNPs that have been reported to be significantly associated with hearing loss in previous GWASs and candidate gene-based association studies.

| No. | Chr. | SNPs | Positions^a^ | PMID number  of the studies | Major alleles/  Minor alleles | Genes^b^ | Diseases | In the present study | | Methods |
| --- | --- | --- | --- | --- | --- | --- | --- | --- | --- | --- |
|  |  |  |  |  |  |  |  | ORs (95% CIs)^c^ | *P* |  |
| 1 | 1 | rs4660468 | 41285087 | 30153627 | T/C | *KCNQ4* | NIHL | 0.98(0.69-1.40) | 0.91 | Candidate gene-based study |
| 2 | 2 | rs1320851 | 66048174 | 24939585 | T/G | *AC007389.1* | ARHL | 0.75 (0.50-1.13) | 0.17 | GWAS |
| 3 | 2 | rs1402901 | 53460592 | 24939585 | A/G | *SCARNA16* | ARHL | 0.53 (0.32-0.87) | 0.01 | GWAS |
| 4 | 2 | rs1872328 | 54395259 | 29358504 | G/A | *ACYP2* | DRHL | NA | NA | Candidate gene-based study |
| 5 | 2 | rs7598759 | 232321956 | 26121033 | C/T | *NCL* | NIHL | 1.20 (0.85-1.68) | 0.29 | GWAS |
| 6 | 3 | rs11928865 | 7155702 | 19047183 | T/A | *GRM7* | ARHL | 0.83(0.49-1.45) | 0.52 | Candidate gene-based study |
| 7 | 5 | rs457717 | 76625147 | 20068591 | A/G | *IQGAP2* | ARHL | 0.69 (0.48-1.00) | 0.05 | GWAS |
| 8 | 6 | rs1043618 | 31783507 | 18813331 | G/A | *HSP70* | NIHL | 0.74 (0.50-1.10) | 0.14 | Candidate gene-based study |
| 9 | 6 | rs10499138 | 128171037 | 20068591 | A/C | *PTPRK* | HL | 1.49 (1.03-2.14) | 0.03 | GWAS |
| 10 | 6 | rs1061581 | 31784586 | 18813331 | G/A | *HSP70* | NIHL | NA | NA | Candidate gene-based study |
| 11 | 6 | rs212769 | 133773548 | 26400775 | C/T | *EYA4* | NIHL | 0.74 (0.36-1.54) | 0.43 | Candidate gene-based study |
| 12 | 6 | rs2227956 | 31778272 | 18813331 | G/A | *HSP70* | NIHL | 0.98(0.63-1.54) | 0.95 | Candidate gene-based study |
| 13 | 6 | rs2763979 | 31794592 | 28182740 | C/T | *HSP70* | NIHL | 1.01(0.70-1.45) | 0.96 | Candidate gene-based study |
| 14 | 6 | rs3777781 | 133568713 | 26400775 | T/A | *EYA4* | NIHL | 0.87 (0.60-1.24) | 0.43 | Candidate gene-based study |
| 15 | 6 | rs3813346 | 133561646 | 27613755 | T/G | *EYA4* | NIHL | 0.92(0.19-0.64) | 0.65 | Candidate gene-based study |
| 16 | 6 | rs4880 | 160113872 | 19493484 | A/G | *SOD2* | NIHL | 0.77(0.44-1.34) | 0.36 | Candidate gene-based study |
| 17 | 6 | rs5746092 | 160114311 | 23500038 | G/C | *SOD2* | ARHL | 0.96(0.67 -1.39) | 0.84 | Candidate gene-based study |
| 18 | 6 | rs9351104 | 86638087 | 24939585 | T/C | *AL353133.1* | ARHL | 0.38 (0.14-1.03) | 0.06 | GWAS |
| 19 | 7 | rs10278194 | 42324206 | 24939585 | A/C | *GLI3* | ARHL | 0.65 (0.42-1.00) | 0.05 | GWAS |
| 20 | 7 | rs2961030 | 52617967 | 24939585 | T/C | *snoU13* | ARHL | 1.43 (0.90-2.27) | 0.13 | GWAS |
| 21 | 8 | rs10955255 | 102536405 | 17921507 | A/G | *GRHL2* | ARHL | 0.78(0.48-1.27) | 0.31 | Candidate gene-based study |
| 22 | 8 | rs1981361 | 102552777 | 17921507 | C/G | *GRHL2* | ARHL | 0.74(0.47-1.17) | 0.19 | Candidate gene-based study |
| 23 | 8 | rs2127034 | 102542398 | 17921507 | C/A | *GRHL2* | ARHL | 0.78(0.48-1.26) | 0.31 | Candidate gene-based study |
| 24 | 10 | rs11250795 | 1804460 | 24939585 | G/A | *ADARB2* | ARHL | 1.43 (1.01-2.02) | 0.05 | GWAS |
| 25 | 10 | rs7095441 | 55871323 | 19183343 | T/C | *PCDH15* | NIHL | NA | NA | Candidate gene-based study |
| 26 | 11 | rs769214 | 34459717 | 25484013 | G/A | *CAT* | NIHL | 1.11(0.765-1.61) | 0.59 | Candidate gene-based study |
| 27 | 11 | rs7943316 | 34460472 | 25484013 | A/T | *CAT* | NIHL | 1.05(0.72-1.51) | 0.82 | Candidate gene-based study |
| 28 | 13 | rs3751385 | 20762956 | 26075227 | A/G | *GJB2* | HL | 1.06(0.75-1.51) | 0.75 | Candidate gene-based study |
| 29 | 13 | rs7329857 | 20762929 | 26075227 | G/A | *GJB2* | NIHL | NA | NA | Candidate gene-based study |
| 30 | 13 | rs7333214 | 20796497 | 26075227 | C/A | *GJB6* | NIHL | 0.71(0.48-1.05) | 0.09 | Candidate gene-based study |
| 31 | 13 | rs7987302 | 20761349 | 26075227 | C/A | *GJB2* | NIHL | NA | NA | Candidate gene-based study |
| 32 | 13 | rs7994748 | 20766130 | 26075227 | G/A | *GJB2* | HL | 1.03(0.72-1.47) | 0.89 | Candidate gene-based study |
| 33 | 13 | rs945369 | 20801273 | 26075227 | T/G | *GJB6* | NIHL | 1.19(0.81-1.74) | 0.38 | Candidate gene-based study |
| 34 | 18 | rs12457810 | 9088652 | 24939585 | T/G | *NDUFV2* | ARHL | 0.50 (0.28-0.90) | 0.02 | Candidate gene-based study |
| 35 | 19 | rs667907 | 50785386 | 19183343 | A/G | *MYH14* | NIHL | 0.91(0.61-1.36) | 0.64 | Candidate gene-based study |
| 36 | 21 | rs10432782 | 33036391 | 19895330 | T/G | *SOD1* | NIHL | 0.83 (0.56-1.22) | 0.34 | Candidate gene-based study |
| 37 | 21 | rs2070424 | 33039320 | 19895330 | A/G | *SOD1* | NIHL | 0.83 (0.56-1.22) | 0.34 | Candidate gene-based study |
| ARHL, age-related hearing loss; CI, confidence interval; Chr., chromosome; DRHL, drug-induced hearing loss; HL, hearing loss; NIHL, noise-induced hearing loss; NA, not available; OR, odds ratio; PMID, PubMed Unique Identifier. SNP, single-nucleotide polymorphism. ^a^Positions based on the Genome Reference Consortium Human Genome Build 37 (GRCh37); ^b^The nearest genes; ^c^ORs and 95% CIs were calculated for the minor allele. | | | | | | | | | | |

**Supplementary Table 4** Pathways enrichment analyses in the discovery stage.

| No. | Pathways | Databases | Beta | SE | *P* | FDR | Significant genes |
| --- | --- | --- | --- | --- | --- | --- | --- |
| 1 | Erbb signaling pathway | KEGG | 0.099 | 0.050 | 0.023 | 0.52 | *PRKCB, PRKCA, NRG1, MAP2K4, NRG3, MAPK9, ERBB4, MAPK10, MAP2K2, TGFA, MAP2K1, MAP2K7, NRG2, AKT1, SHC4, AKT3, AREG, GSK3B, KRAS, SHC3, NCK2, CAMK2B, CAMK2D, CRK, PIK3CB, PLCG2, PIK3R1* |
| 2 | Wnt signaling pathway | KEGG | 0.082 | 0.040 | 0.037 | 0.49 | *LRP5, LRP6, PPP3R2, PPP3CC, VANGL1, SENP2, FZD8, PRICKLE1, WNT9B, WNT9A, WNT2B, DKK2, AXIN1, AXIN2, NFAT5, CSNK2A2, CSNK1A1, BTRC, WNT5B, NLK, CAMK2B, CAMK2D, PRKACA, PRKACB, WNT16, DAAM1, CCND2, NFATC2, NFATC1, CCND3, PLCB1, PRKCB, RKCA, PLCB4, PRICKLE2, FRAT2, MAPK9, MAPK10, WNT3A, RAC2, CXXC4, CUL1, SMAD3, TCF7L2, GSK3B, WNT2, WNT3, WNT6, PPP2CB, NKD2, PPP2R5E, PPP2R5C, SFRP4* |
| 3 | Intraflagellar transport | Reactome | -0.085 | 0.085 | 0.044 | 0.66 | *IFT80, KIFAP3, DYNLL1, TUBB1, TTC26, KIF17, WDR35, IFT43, IFT81, TUBB6, DYNC2H1, TUBA3C,* |
| 4 | Hedgehog signaling pathway | KEGG | 0.061 | 0.056 | 0.050 | 0.70 | *HHIP, PTCH2, WNT3A, WNT9B, WNT9A, LRP2, WNT2B, SMO, CSNK1G3, CSNK1A1, BMP8A, GSK3B, BTRC, WNT2, WNT3, WNT6, WNT5B, PRKACA, PRKACB, SUFU, BMP2, GLI2, GLI3, PTCH1, WNT16, BMP5, BMP6* |

Multi-marker Analysis of GenoMic Annotation (MAGMA) was used to perform the pathway-based associations in the genome-wide association study (GWAS) stage in this study. Multiple testing correction was performed using the Benjamini-Hochberg (BH) method. MAGMA software and auxiliary files can be downloaded from <http://ctglab.nl/software/magma>. FDR, false discovery rate; SE, standard error.

**Supplementary Table 5** Summary of the 29 top significantly associated SNPs in the discovery stage.

| No. | Chr. | SNPs | Positions^a^ | Alleles^b^ | Minor hom / Het / Major hom^c^ | | Genes^d^ | ORs (95% CIs)^e^ | *P*^e^ | ORs (95% CIs)^f^ | *P*^f^ | eQTL genes |
| --- | --- | --- | --- | --- | --- | --- | --- | --- | --- | --- | --- | --- |
|  |  |  |  |  | Cases | Controls |  |  |  |  |  |  |
| 1 | 3 | rs73120118 | 78003245 | A/G | 13/48/27 | 6/77/119 | *LOC105377171* | 3.16 (2.02-4.93) | 4.06E-07 | 3.16 (2.02-4.93) | 4.22E-07 |  |
| 2 | 11 | rs76721811 | 69905706 | A/C | 6/34/45 | 2/32/156 | *RP11-626H12.3* | 3.72 (2.22-6.24) | 6.05E-07 | 3.75 (2.23-6.30) | 5.72E-07 |  |
| 3 | 20 | rs804692 | 22250088 | A/G | 33/41/13 | 27/117/63 | *LOC105372561* | 2.61 (1.72-3.94) | 5.67E-06 | 2.61 (1.72-3.95) | 5.77E-06 |  |
| 4 | 7 | rs10081191 | 158322806 | A/C | 16/45/24 | 10/78/105 | *PTPRN2* | 2.58 (1.70-3.92) | 8.74E-06 | 2.58 (1.70-3.93) | 8.63E-06 | *WDR60* |
| 5 | 3 | rs9858841 | 61229611 | C/T | 16/38/29 | 10/70/118 | *FHIT* | 2.50 (1.66-3.75) | 1.05E-05 | 2.51 (1.67-3.77) | 9.74E-06 |  |
| 6 | 18 | rs7234759 | 5465865 | C/T | 4/23/54 | 26/101/67 | *EPB41L3* | 0.35 (0.22-0.56) | 1.14E-05 | 0.34 (0.21-0.56) | 1.17E-05 |  |
| 7 | 10 | rs77211069 | 67047391 | G/A | 1/22/66 | 0/14/194 | *RP11-428G2.1* | 4.92 (2.40-10.10) | 1.39E-05 | 4.91 (2.39-10.09) | 1.43E-05 |  |
| 8 | 8 | rs4733466 | 30130848 | G/A | 9/40/36 | 4/60/133 | *DCTN6* | 2.69 (1.71-4.23) | 1.95E-05 | 2.69 (1.71-4.23) | 2.00E-05 |  |
| 9 | 9 | rs59776070 | 7860699 | C/G | 4/38/45 | 5/41/160 | *C9orf123* | 2.81 (1.75-4.52) | 2.10E-05 | 2.81 (1.74-4.52) | 2.13E-05 |  |
| 10 | 14 | rs8004456 | 92997183 | A/C | 13/43/32 | 7/75/127 | *RIN3* | 2.46 (1.63-3.73) | 2.12E-05 | 2.46 (1.62-3.73) | 2.16E-05 |  |
| 11 | 15 | rs58797922 | 87138950 | T/G | 28/43/15 | 23/101/71 | *AGBL1* | 2.39 (1.60-3.58) | 2.17E-05 | 2.39 (1.60-3.58) | 2.18E-05 |  |
| 12 | 1 | rs11206087 | 53544289 | T/C | 8/42/38 | 4/63/137 | *PODN* | 2.61 (1.66-4.10) | 3.03E-05 | 2.63 (1.67-4.13) | 2.80E-05 |  |
| 13 | 7 | rs11971906 | 62464272 | T/G | 7/37/43 | 4/55/148 | *U6* | 2.63 (1.66-4.17) | 3.71E-05 | 2.63 (1.66-4.16) | 3.95E-05 | *RP11-196D18.1* |
| 14 | 4 | rs6448586 | 28556177 | T/A | 5/43/37 | 6/49/145 | *LOC105374557* | 2.62 (1.65-4.16) | 4.32E-05 | 2.63 (1.65-4.17) | 4.56E-05 |  |
| 15 | 11 | rs592036 | 125850139 | C/A | 9/23/56 | 3/32/173 | *CDON* | 2.67 (1.67-4.27) | 4.40E-05 | 2.67 (1.67-4.28) | 4.56E-05 |  |
| 16 | 10 | rs35827735 | 131824295 | A/G | 1/21/67 | 0/16/193 | *RP11-500G10.1* | 4.31 (2.12-8.78) | 5.61E-05 | 4.46 (2.15-9.23) | 5.71E-05 |  |
| 17 | 2 | rs6710946 | 212295875 | C/T | 16/45/28 | 15/79/115 | *ERBB4* | 2.19 (1.49-3.23) | 6.34E-05 | 2.22 (1.51-3.26) | 5.44E-05 |  |
| 18 | 13 | rs79939311 | 44552505 | A/G | 1/29/58 | 1/27/173 | *LOC107984576* | 3.30 (1.84-5.93) | 6.50E-05 | 3.31 (1.84-5.95) | 6.28E-05 |  |
| 19 | 1 | rs58822919 | 233748783 | A/C | 3/33/47 | 4/32/161 | *KCNK1* | 2.81 (1.69-4.67) | 6.82E-05 | 2.81 (1.69-4.67) | 6.94E-05 |  |
| 20 | 7 | rs35075890 | 70178230 | G/A | 1/20/67 | 0/13/193 | *AUTS2* | 4.53 (2.15-9.54) | 7.02E-05 | 4.92 (2.29-10.55) | 4.31E-05 |  |
| 21 | 11 | rs259874 | 61811441 | G/A | 18/50/21 | 20/90/99 | *RP11-810P12.5* | 2.19 (1.48-3.22) | 7.63E-05 | 2.22 (1.50-3.28) | 6.59E-05 |  |
| 22 | 12 | rs4262771 | 9479318 | C/G | 18/46/18 | 22/82/94 | *SNORA75* | 2.19 (1.48-3.24) | 8.72E-05 | 2.19 (1.48-3.24) | 8.93E-05 | *A2MP1 DDX12P* |
| 23 | 5 | rs9325087 | 147565858 | T/C | 10/37/37 | 53/99/43 | *SPINK14* | 0.46 (0.31-0.67) | 8.89E-05 | 0.45 (0.30-0.67) | 7.68E-05 | *FBXO38*  SPINK6 |
| 24 | 3 | rs56328361 | 142357698 | T/C | 2/19/63 | 1/15/181 | *PLS1* | 3.98 (2.00-7.96) | 8.92E-05 | 3.95 (1.98-7.90) | 9.96E-05 |  |
| 25 | 7 | rs76547605 | 153960330 | C/A | 7/33/48 | 4/45/159 | *DPP6* | 2.48 (1.57-3.91) | 9.05E-05 | 2.48 (1.58-3.92) | 8.92E-05 |  |
| 26 | 21 | rs2826626 | 22341330 | A/C | 7/35/41 | 1/55/131 | *NCAM2* | 2.59 (1.61-4.19) | 9.93E-05 | 2.59 (1.60-4.19) | 1.02E-04 |  |
| 27 | 7 | rs6951489 | 76639839 | G/A | 3/32/47 | 31/94/68 | *UPK3BP1* | 0.42 (0.27-0.65) | 1.00E-04 | 0.42 (0.27-0.65) | 9.33E-05 | *PMS2P1 UPK3BP1* |
| 28 | 20 | rs1760055 | 60763556 | A/C | 21/44/22 | 15/91/80 | *MTG2* | 2.24 (1.49-3.37) | 1.00E-04 | 2.25 (1.50-3.37) | 9.71E-05 | *MTG2* |
| 29 | 4 | rs10009367 | 181203371 | C/G | 10/47/32 | 11/71/126 | *LOC105377567* | 2.23 (1.49-3.35) | 1.00E-04 | 2.24 (1.49-3.37) | 1.04E-04 |  |
| CI, confidence interval; Chr., chromosome; MAF, Minor allele frequency; OR, odds ratio; SNP, single-nucleotide polymorphism. ^a^Positions based on the Genome Reference Consortium Human Genome Build 37 (GRCh37); ^b^Minor allele/major allele; ^c^Counts of Minor allele homozygote / heterozygote / major allele homozygote; ^d^Genes or nearest genes; ^e^ORs and 95% CIs were calculated after adjusting for age and noise exposure time for the minor allele. ^f^ORs, 95% CIs and *P* values were calculated after adjusting for age and noise exposure time and the significant principal component (PC) 1. | | | | | | | | | | | | |

**Supplementary Table 6** Primers used for SNPs genotyping in the replication stage.

| No. | SNPs | Primers | Sequences (5'→3') |
| --- | --- | --- | --- |
| 1 | rs73120118 | Forward | ACGTTGGATGAGTCTATGACAACGACTGCC |
|  |  | Reverse | ACGTTGGATGGCTTTTTAATGGCTAGGGAG |
|  |  | Extend | CATGCCATTTACAGAGAAATTGAAAAA |
| 2 | rs76721811 | Forward | ACGTTGGATGGTTGGAGTAAATCCACAAGC |
|  |  | Reverse | ACGTTGGATGACCTCTCAATGTTCTGCCTG |
|  |  | Extend | AGGCCTCCTCCTTTTCCTAC |
| 3 | rs804692 | Forward | ACGTTGGATGCACCACGCCTGGCTAATTTT |
|  |  | Reverse | ACGTTGGATGTGGCCTGATTAAAAATGGAG |
|  |  | Extend | GTGGACTGATTAAAAATGGAGCAAGGT |
| 4 | rs10081191 | Forward | ACGTTGGATGGAGATCCATTCCTAGGACAG |
|  |  | Reverse | ACGTTGGATGTAGCCAGGGAACCAAATCAC |
|  |  | Extend | GACAGGCTGACCCCA |
| 5 | rs9858841 | Forward | ACGTTGGATGTGACAGCTTTGTGATGACGC |
|  |  | Reverse | ACGTTGGATGAGGTAAAAGGTCTGAGTGGG |
|  |  | Extend | GCTTCGTGATGACGCTTTTCTTTCA |
| 6 | rs7234759 | Forward | ACGTTGGATGATGGAGTGGAATGGGAGAAC |
|  |  | Reverse | ACGTTGGATGTGTAGATACCCTAACAGTTG |
|  |  | Extend | AAGTCTCATTTAATTCATGAAATTCC |
| 7 | rs77211069 | Forward | ACGTTGGATGATATCAGGGTTGAAGTGTCC |
|  |  | Reverse | ACGTTGGATGCCGTTCCTCAACGGAGAAAA |
|  |  | Extend | AAGGGTTTGCCACATTAA |
| 8 | rs4733466 | Forward | ACGTTGGATGTCTAGGCTAGAGGAAGAGAG |
|  |  | Reverse | ACGTTGGATGAAAGTAGAGCAGGGTGCAAG |
|  |  | Extend | TAAGCAGCATATTACTCCT |
| 9 | rs59776070 | Forward | ACGTTGGATGGGAAGTTGATAACATGCTTG |
|  |  | Reverse | ACGTTGGATGTCTGTTTGTCCTTCTGCCTC |
|  |  | Extend | TTTCCTTCTGCCTCAGAAATATTATG |
| 10 | rs8004456 | Forward | ACGTTGGATGAACCTCTGCAACCTTCCAAG |
|  |  | Reverse | ACGTTGGATGCTGGGTTGTCCACATAGAAC |
|  |  | Extend | GCCACTGAGGGGATGA |
| 11 | rs58797922 | Forward | ACGTTGGATGCCCACTGCACAGATAAAACC |
|  |  | Reverse | ACGTTGGATGTATCTATCACTCCGCTAGCC |
|  |  | Extend | TCTCTAGTAGTATTGCAGTAGAAAAAGT |
| 12 | rs11206087 | Forward | ACGTTGGATGTTGCACACGGTGCACCTGTA |
|  |  | Reverse | ACGTTGGATGTGGTTGTGCAGGATCATGAG |
|  |  | Extend | CACTGTGCACCTGTACAACAA |
| 13 | rs11971906 | Forward | ACGTTGGATGAAGGCCCTTCAAGCTAGCAC |
|  |  | Reverse | ACGTTGGATGGGGAGAACTCATTTTACCAC |
|  |  | Extend | GCAATGATACAGCCTTATTTGTC |
| 14 | rs6448586 | Forward | ACGTTGGATGTAGTGGGTTGAAGAGTGTCC |
|  |  | Reverse | ACGTTGGATGATGTCATCTCAACCCTTACC |
|  |  | Extend | CAACCTCATTATGTGATCTT |
| 15 | rs592036 | Forward | ACGTTGGATGTTTCTAGTGCAGAGAACCCC |
|  |  | Reverse | ACGTTGGATGGAGATAACTAGTTGATAGCG |
|  |  | Extend | GGTATAGCGTTTAATTTTTGAAATGG |
| 16 | rs35827735 | Forward | ACGTTGGATGATTTGCGGTTGAAAGGCGAG |
|  |  | Reverse | ACGTTGGATGCTGATGAAGCACAGGCAGG |
|  |  | Extend | ACGAGATGTGGACAGCAGCA |
| 17 | rs6710946 | Forward | ACGTTGGATGCTTCCAGGTACATCATTCCC |
|  |  | Reverse | ACGTTGGATGCCAACTGAAGGCTAAGAAAC |
|  |  | Extend | CCTCCGTTAATGCCCAGGTTTT |
| 18 | rs79939311 | Forward | ACGTTGGATGTGGCTTAGCATTGTGTCCAG |
|  |  | Reverse | ACGTTGGATGATTTCCAGAAAGGGTCCTTG |
|  |  | Extend | GCAGTCTCTTTCTTTCACTCAAT |
| 19 | rs58822919 | Forward | ACGTTGGATGGTCCCACATGTGTATGCACT |
|  |  | Reverse | ACGTTGGATGCAAAGACTGGTCATAGACGG |
|  |  | Extend | AACATGTGTATGCACTATATTATACAA |
| 20 | rs35075890 | Forward | ACGTTGGATGCATCTGTGTGAAGGGCTATC |
|  |  | Reverse | ACGTTGGATGTAATCACGGCACATTTGCCC |
|  |  | Extend | CCGGCTATCACAGCACAC |
| 21 | rs259874 | Forward | ACGTTGGATGGTTCTTCAGCCCAGGGATTC |
|  |  | Reverse | ACGTTGGATGGTCCAAATACTGCCTTTCCC |
|  |  | Extend | GGACTTCTGAGAAGCATTGAGTCCA |
| 22 | rs4262771 | Forward | ACGTTGGATGGAGTCTTCCAGTTGTACAGG |
|  |  | Reverse | ACGTTGGATGATAAGCACTCAGGCCCATTC |
|  |  | Extend | GGTCAGAAGTAGAGAAGC |
| 23 | rs9325087 | Forward | ACGTTGGATGGCAATGAGAAAAAGGGACAC |
|  |  | Reverse | ACGTTGGATGTGACGAAGGAGGAGATTGAG |
|  |  | Extend | GAGAAAAAGGGACACATAAGA |
| 24 | rs56328361 | Forward | ACGTTGGATGTAATGGTGCGATCTCAGCTC |
|  |  | Reverse | ACGTTGGATGGCCTATAATCCCACCTACTC |
|  |  | Extend | TCAAGTGATTCTCCTGC |
| 25 | rs76547605 | Forward | ACGTTGGATGGGGAAAGACTCAACAGAAAC |
|  |  | Reverse | ACGTTGGATGCCTGATTTACTCCTGAGCTG |
|  |  | Extend | GCTCCTTTCAGGAGACTAAGC |
| 26 | rs2826626 | Forward | ACGTTGGATGTCTTTAAGCATGCACTCTGG |
|  |  | Reverse | ACGTTGGATGGCAGTGTGCTGAAGAATATC |
|  |  | Extend | TGCTGCTTTCTTGCAT |
| 27 | rs6951489 | Forward | ACGTTGGATGTCTCCATCATCCCTGCAATC |
|  |  | Reverse | ACGTTGGATGTTCAGCTAGAGAGGAACCAC |
|  |  | Extend | CGCCTCCCTGCAATCCCCAAGCAAGA |
| 28 | rs1760055 | Forward | ACGTTGGATGTATTATAGGCATGCACCACC |
|  |  | Reverse | ACGTTGGATGTAGCGAGACCTTGTGTCTAC |
|  |  | Extend | ACCACCATGTCCAGCTA |
| 29 | rs10009367 | Forward | ACGTTGGATGCTGGATACAGATAGTGGGAG |
|  |  | Reverse | ACGTTGGATGCTCATTCACTACATTGTAAGC |
|  |  | Extend | CATTGTAAGCTTTTTTGATGTTGA |
| SNP, single nucleotide polymorphism. Primers were used for genotyping using Sequenom MassArray System (Sequenom Inc., USA). PCR was performed with an initial 2 minutes (min) at 50°C and 10 min at 95°C, followed by 40 cycles of 15 seconds at 95°C and 1 min at 60°C. In the replication stage, 29 SNPs were selected for genotyping. Finally, among these 29 SNPs, two SNPs (rs35075890 and rs10081191) survived in the replication stage. | | | |

**Supplementary Table 7** Summary of the genetic association results for the 29 SNPs in the replication stage.

| No. | Chr. | SNPs | Positions^a^ | Alleles^b^ | Minor hom / Het / Major hom^c^ | | Genes^d^ | ORs (95% CIs)^e^ | *P* |
| --- | --- | --- | --- | --- | --- | --- | --- | --- | --- |
|  |  |  |  |  | Cases | Controls |  |  |  |
| 1 | 3 | rs73120118 | 78003245 | A/G | 5/18/28 | 33/140/184 | *LOC105377171* | 0.95 (0.60-1.50) | 0.82 |
| 2 | 11 | rs76721811 | 69905706 | A/C | 1/13/37 | 11/97/239 | *RP11-626H12.3* | 0.87 (0.49-1.56) | 0.65 |
| 3 | 20 | rs804692 | 22250088 | A/G | 11/24/15 | 89/169/92 | *LOC105372561* | 0.89 (0.59-1.35) | 0.59 |
| 4 | 7 | rs10081191 | 158322806 | A/C | 12/19/22 | 37/142/178 | *PTPRN2* | 1.52 (1.02-2.27) | 0.020 |
| 5 | 3 | rs9858841 | 61229611 | C/T | 3/22/25 | 33/129/186 | *FHIT* | 1.00 (0.63-1.58) | 1.00 |
| 6 | 18 | rs7234759 | 5465865 | C/T | 6/22/23 | 37/152/163 | *EPB41L3* | 1.05 (0.68-1.63) | 0.81 |
| 7 | 10 | rs77211069 | 67047391 | G/A | 1/5/45 | 1/21/335 | *RP11-428G2.1* | 1.95 (0.84-4.50) | 0.12 |
| 8 | 8 | rs4733466 | 30130848 | G/A | 3/21/27 | 26/118/211 | *DCTN6* | 1.11 (0.70-1.76) | 0.66 |
| 9 | 9 | rs59776070 | 7860699 | C/G | 2/16/33 | 10/121/217 | *C9orf123* | 0.93 (0.54-1.61) | 0.81 |
| 10 | 14 | rs8004456 | 92997183 | A/C | 1/20/30 | 25/117/212 | *RIN3* | 0.86 (0.52-1.41) | 0.54 |
| 11 | 15 | rs58797922 | 87138950 | T/G | 12/23/16 | 60/176/118 | *AGBL1* | 1.19 (0.78-1.81) | 0.42 |
| 12 | 1 | rs11206087 | 53544289 | T/C | 1/17/33 | 15/108/229 | *PODN* | 0.94 (0.55-1.60) | 0.82 |
| 13 | 7 | rs11971906 | 62464272 | T/G | 0/15/36 | 10/113/233 | *U6* | 0.74 (0.41-1.35) | 0.33 |
| 14 | 4 | rs6448586 | 28556177 | T/A | 3/12/36 | 24/117/212 | *LOC105374557* | 0.73 (0.43-1.23) | 0.23 |
| 15 | 11 | rs592036 | 125850139 | C/A | 2/10/39 | 10/91/252 | *CDON* | 0.83 (0.46-1.50) | 0.53 |
| 16 | 10 | rs35827735 | 131824295 | A/G | 0/8/43 | 2/41/314 | *RP11-500G10.1* | 1.29 (0.59-2.80) | 0.53 |
| 17 | 2 | rs6710946 | 212295875 | C/T | 3/29/19 | 36/160/158 | *ERBB4* | 1.08 (0.68-1.70) | 0.75 |
| 18 | 13 | rs79939311 | 44552505 | A/G | 1/9/41 | 3/68/276 | *LOC107984576* | 0.96 (0.48-1.90) | 0.90 |
| 19 | 1 | rs58822919 | 233748783 | A/C | 2/10/39 | 8/86/255 | *KCNK1* | 0.99 (0.54-1.81) | 0.98 |
| 20 | 7 | rs35075890 | 70178230 | G/A | 0/12/39 | 1/40/313 | *AUTS2* | 2.21 (1.09-4.47) | 0.0035 |
| 21 | 11 | rs259874 | 61811441 | G/A | 9/25/17 | 43/160/154 | *RP11-810P12.5* | 1.41 (0.92-2.16) | 0.12 |
| 22 | 12 | rs4262771 | 9479318 | C/G | 5/19/27 | 38/166/150 | *SNORA75* | 0.77 (0.48-1.22) | 0.26 |
| 23 | 5 | rs9325087 | 147565858 | T/C | 16/16/19 | 73/168/108 | *SPINK14* | 1.09 (0.73-1.63) | 0.68 |
| 24 | 7 | rs76547605 | 153960330 | C/A | 1/12/38 | 10/95/248 | *DPP6* | 0.81 (0.45-1.48) | 0.50 |
| 25 | 21 | rs2826626 | 22341330 | A/C | 2/17/32 | 13/121/214 | *NCAM2* | 0.97 (0.58-1.64) | 0.91 |
| 26 | 7 | rs6951489 | 76639839 | G/A | 4/21/26 | 52/113/189 | *UPK3BP1* | 0.92 (0.61-1.40) | 0.71 |
| 27 | 20 | rs1760055 | 60763556 | A/C | 6/24/21 | 69/170/118 | *MTG2* | 0.74 (0.48-1.15) | 0.18 |
| 28 | 4 | rs10009367 | 181203371 | C/G | 2/24/25 | 22/130/205 | *LOC105377567* | 1.18 (0.73-1.90) | 0.50 |
| CI, confidence interval; Chr., chromosome; MAF, Minor allele frequency; OR, odds ratio; SNP, single-nucleotide polymorphism. ^a^Positions based on the Genome Reference Consortium Human Genome Build 37 (GRCh37); ^b^Minor allele/major allele; ^c^Counts of Minor allele homozygote / heterozygote / major allele homozygote; ^d^Genes or nearest genes; ^e^ORs and 95% CIs were calculated for the minor allele; Genotyping for rs56328361 failed in this study. | | | | | | | | | |
|  |  |  |  |  |  |  |  |  |  |
|  |  |  |  |  |  |  |  |  |  |
|  |  |  |  |  |  |  |  |  |  |

**Supplementary Table 8** Stratification analyses of rs35075890 and rs10081191 by age.

| Categories | Genotypes | rs35075890 | | | | | |
| --- | --- | --- | --- | --- | --- | --- | --- |
|  |  | Discovery  stage, n | | Replication  stage, n | | Overall, n | |
|  |  | Cases | Controls | Cases | Controls | Cases | Controls |
| Age, years |  |  |  |  |  |  |  |
| ≤ 23 | GG | 0 | 0 | 0 | 1 | 0 | 1 |
|  | GA | 8 | 6 | 3 | 16 | 11 | 22 |
|  | AA | 26 | 97 | 10 | 118 | 36 | 215 |
| ORs (95% CIs) |  | 4.97 (1.59-15.61) | | 1.80 (0.50-6.46) | | 2.54 (1.18-5.47) | |
| *P* |  | 5.97E-03 | | 0.37 | | 0.020 | |
| > 23 | GG | 1 | 0 | 0 | 0 | 1 | 0 |
|  | GA | 12 | 7 | 9 | 24 | 36 | 31 |
|  | AA | 41 | 96 | 29 | 195 | 70 | 291 |
| ORs (95% CIs) |  | 4.25 (1.62-11.13) | | 2.52 (1.07-5.96) | | 2.98 (1.65-5.38) | |
| *P* |  | 0.0033 | | 0.030 | | 0.00029 | |
| *P*_heterogeneity_ |  | 0.86 | | 0.85 | | 0.99 | |
| Categories | Genotypes | rs10081191 | | | | | |
|  |  | Discovery  stage, n | | Replication  stage, n | | Overall, n | |
|  |  | Cases | Controls | Cases | Controls | Cases | Controls |
| Age, years |  |  |  |  |  |  |  |
| ≤ 23 | CC | 7 | 52 | 7 | 69 | 14 | 121 |
|  | AC | 22 | 41 | 4 | 53 | 26 | 94 |
|  | AA | 5 | 4 | 2 | 12 | 7 | 16 |
| ORs (95% CIs) |  | 3.34 (1.66-6.72) | | 1.10 (0.47-2.57) | | 2.05 (1.27-3.31) | |
| *P* |  | 0.00074 | | 0.83 | | 0.0034 | |
| > 23 | CC | 17 | 53 | 15 | 109 | 32 | 162 |
|  | AC | 23 | 37 | 15 | 89 | 38 | 126 |
|  | AA | 11 | 6 | 10 | 25 | 21 | 31 |
| ORs (95% CIs) |  | 2.25 (1.34-3.79) | | 1.63 (1.03-2.60) | | 1.79 (1.29-2.49) | |
| *P* |  | 0.0022 | | 0.039 | | 0.00054 | |
| *P*_heterogeneity_ |  | 0.38 | | 0.16 | | 0.57 | |
| CI, confidence interval; OR, odds ratio. The *P* values, ORs and 95% CIs were calculated under an additive model by logistic regression while adjusting for age. Using the meta-analysis helper (METAL) software to calculate the heterogeneity of the population in the discovery stage and the replication stage. *P*_heterogeneity_ value of less than 0.05 was considered to be statistically significant. | | | | | | | |

**Supplementary Table 9** The eQTL analyses of rs35075890 and rs10081191 in 13 types of human brain tissues from GTEx.

| (A) The eQTL analyses of rs35075890 in 13 types of human brain tissues from GTEx. | | | | |
| --- | --- | --- | --- | --- |
| Genes | SNPs | *P* | NES | Tissues |
| *AUTS2* | rs35075890 | 0.19 | 0.11 | Brain_Amygdala |
| *AUTS2* | rs35075890 | 0.47 | 0.05 | Brain_Anterior_cingulate_cortex_BA24 |
| *AUTS2* | rs35075890 | 0.67 | 0.02 | Brain_Caudate _basal ganglia |
| *AUTS2* | rs35075890 | 0.87 | -0.01 | Brain_Cerebellar_Hemisphere |
| *AUTS2* | rs35075890 | 0.15 | 0.09 | Brain_Cerebellum |
| *AUTS2* | rs35075890 | 0.29 | 0.07 | Brain_Cortex |
| *AUTS2* | rs35075890 | 2.90E-03 | 0.20 | Brain_Frontal_Cortex_BA9 |
| *AUTS2* | rs35075890 | 7.50E-03 | 0.17 | Brain_Hippocampus |
| *AUTS2* | rs35075890 | 2.00E-02 | 0.17 | Brain_Hypothalamus |
| *AUTS2* | rs35075890 | 0.40 | -0.05 | Brain_Nucleus_accumbens_basal_ganglia |
| *AUTS2* | rs35075890 | 0.38 | 0.06 | Brain_Putamen_basal_ganglia |
| *AUTS2* | rs35075890 | 0.96 | 0.00 | Brain_Spinal_cord_cervical_c-1 |
| *AUTS2* | rs35075890 | 0.77 | 0.02 | Brain_Substantia_nigra |
| *WBSCR17* | rs35075890 | 0.021 | 0.09 | Brain_Amygdala |
| *WBSCR17* | rs35075890 | 0.29 | 0.03 | Brain_Anterior_cingulate_cortex_BA24 |
| *WBSCR17* | rs35075890 | 0.88 | 0.00 | Brain_Caudate _basal ganglia |
| *WBSCR17* | rs35075890 | 0.49 | -0.05 | Brain_Cerebellar_Hemisphere |
| *WBSCR17* | rs35075890 | 0.088 | -0.11 | Brain_Cerebellum |
| *WBSCR17* | rs35075890 | 0.23 | 0.02 | Brain_Cortex |
| *WBSCR17* | rs35075890 | 0.49 | 0.02 | Brain_Frontal_Cortex_BA9 |
| *WBSCR17* | rs35075890 | 0.28 | -0.03 | Brain_Hippocampus |
| *WBSCR17* | rs35075890 | 0.16 | -0.06 | Brain_Hypothalamus |
| *WBSCR17* | rs35075890 | 0.35 | -0.03 | Brain_Nucleus_accumbens_basal_ganglia |
| *WBSCR17* | rs35075890 | 0.34 | 0.03 | Brain_Putamen_basal_ganglia |
| *WBSCR17* | rs35075890 | 0.83 | -0.01 | Brain_Spinal_cord_cervical_c-1 |
| *WBSCR17* | rs35075890 | 0.78 | 0.02 | Brain_Substantia_nigra |
| (B) The eQTL analyses of rs10081191 in 13 types of human brain tissues from GTEx. | | | | |
| Genes | SNPs | *P* | NES | Tissues |
| *ESYT2* | rs10081191 | 0.90 | -0.01 | Brain_Amygdala |
| *ESYT2* | rs10081191 | 0.57 | 0.05 | Brain_Anterior_cingulate_cortex_BA24 |
| *ESYT2* | rs10081191 | 0.41 | -0.05 | Brain_Caudate _basal ganglia |
| *ESYT2* | rs10081191 | 0.98 | 0.00 | Brain_Cerebellar_Hemisphere |
| *ESYT2* | rs10081191 | 0.21 | 0.07 | Brain_Cerebellum |
| *ESYT2* | rs10081191 | 0.84 | -0.01 | Brain_Cortex |
| *ESYT2* | rs10081191 | 0.76 | -0.02 | Brain_Frontal_Cortex_BA9 |
| *ESYT2* | rs10081191 | 0.74 | 0.02 | Brain_Hippocampus |
| *ESYT2* | rs10081191 | 0.91 | -0.01 | Brain_Hypothalamus |
| *ESYT2* | rs10081191 | 0.95 | 0.00 | Brain_Nucleus_accumbens_basal_ganglia |
| *ESYT2* | rs10081191 | 0.15 | -0.08 | Brain_Putamen_basal_ganglia |
| *ESYT2* | rs10081191 | 0.16 | -0.09 | Brain_Spinal_cord_cervical_c-1 |
| *ESYT2* | rs10081191 | 0.40 | 0.10 | Brain_Substantia_nigra |
| *NCAPG2* | rs10081191 | 0.66 | 0.04 | Brain_Amygdala |
| *NCAPG2* | rs10081191 | 0.81 | 0.02 | Brain_Anterior_cingulate_cortex_BA24 |
| *NCAPG2* | rs10081191 | 0.85 | -0.01 | Brain_Caudate _basal ganglia |
| *NCAPG2* | rs10081191 | 0.23 | 0.09 | Brain_Cerebellar_Hemisphere |
| *NCAPG2* | rs10081191 | 0.86 | 0.01 | Brain_Cerebellum |
| *NCAPG2* | rs10081191 | 0.67 | -0.03 | Brain_Cortex |
| *NCAPG2* | rs10081191 | 0.35 | 0.06 | Brain_Frontal_Cortex_BA9 |
| *NCAPG2* | rs10081191 | 0.67 | 0.03 | Brain_Hippocampus |
| *NCAPG2* | rs10081191 | 0.26 | -0.08 | Brain_Hypothalamus |
| *NCAPG2* | rs10081191 | 0.13 | 0.09 | Brain_Nucleus_accumbens_basal_ganglia |
| *NCAPG2* | rs10081191 | 0.40 | 0.17 | Brain_Putamen_basal_ganglia |
| *NCAPG2* | rs10081191 | 0.15 | 0.15 | Brain_Spinal_cord_cervical_c-1 |
| *NCAPG2* | rs10081191 | 0.52 | 0.06 | Brain_Substantia_nigra |
| *PTPRN2* | rs10081191 | 2.00E-04 | 0.12 | Brain_Amygdala |
| *PTPRN2* | rs10081191 | 1.00E-04 | 0.14 | Brain_Anterior_cingulate_cortex_BA24 |
| *PTPRN2* | rs10081191 | 3.10E-02 | 0.06 | Brain_Caudate _basal ganglia |
| *PTPRN2* | rs10081191 | 2.00E-02 | 0.13 | Brain_Cerebellar_Hemisphere |
| *PTPRN2* | rs10081191 | 0.67 | 0.02 | Brain_Cerebellum |
| *PTPRN2* | rs10081191 | 3.20E-07 | 0.16 | Brain_Cortex |
| *PTPRN2* | rs10081191 | 4.30E-07 | 0.17 | Brain_Frontal_Cortex_BA9 |
| *PTPRN2* | rs10081191 | 0.25 | 0.03 | Brain_Hippocampus |
| *PTPRN2* | rs10081191 | 2.50E-02 | 0.06 | Brain_Hypothalamus |
| *PTPRN2* | rs10081191 | 1.30E-03 | 0.10 | Brain_Nucleus_accumbens_basal_ganglia |
| *PTPRN2* | rs10081191 | 1.20E-02 | 0.07 | Brain_Putamen_basal_ganglia |
| *PTPRN2* | rs10081191 | 0.73 | -0.02 | Brain_Spinal_cord_cervical_c-1 |
| *PTPRN2* | rs10081191 | 0.25 | 0.04 | Brain_Substantia_nigra |
| *VIPR2* | rs10081191 | 0.32 | 0.08 | Brain_Amygdala |
| *VIPR2* | rs10081191 | 0.74 | 0.03 | Brain_Anterior_cingulate_cortex_BA24 |
| *VIPR2* | rs10081191 | 0.98 | 0.00 | Brain_Caudate _basal ganglia |
| *VIPR2* | rs10081191 | 0.23 | -0.10 | Brain_Cerebellar_Hemisphere |
| *VIPR2* | rs10081191 | 0.33 | -0.08 | Brain_Cerebellum |
| *VIPR2* | rs10081191 | 0.34 | -0.16 | Brain_Cortex |
| *VIPR2* | rs10081191 | 0.10 | -0.14 | Brain_Frontal_Cortex_BA9 |
| *VIPR2* | rs10081191 | 0.22 | 0.10 | Brain_Hippocampus |
| *VIPR2* | rs10081191 | 0.76 | 0.02 | Brain_Hypothalamus |
| *VIPR2* | rs10081191 | 0.17 | -0.11 | Brain_Nucleus_accumbens_basal_ganglia |
| *VIPR2* | rs10081191 | 0.42 | -0.06 | Brain_Putamen_basal_ganglia |
| *VIPR2* | rs10081191 | 0.22 | -0.12 | Brain_Spinal_cord_cervical_c-1 |
| *VIPR2* | rs10081191 | 0.33 | 0.10 | Brain_Substantia_nigra |
| *WDR60* | rs10081191 | 4.20E-04 | 0.27 | Brain_Amygdala |
| *WDR60* | rs10081191 | 0.10 | 0.13 | Brain_Anterior_cingulate_cortex_BA24 |
| *WDR60* | rs10081191 | 2.30E-02 | 0.12 | Brain_Caudate _basal ganglia |
| *WDR60* | rs10081191 | 0.15 | 0.13 | Brain_Cerebellar_Hemisphere |
| *WDR60* | rs10081191 | 0.12 | 0.14 | Brain_Cerebellum |
| *WDR60* | rs10081191 | 0.51 | 0.04 | Brain_Cortex |
| *WDR60* | rs10081191 | 0.52 | 0.04 | Brain_Frontal_Cortex_BA9 |
| *WDR60* | rs10081191 | 0.23 | 0.07 | Brain_Hippocampus |
| *WDR60* | rs10081191 | 9.60E-03 | 0.13 | Brain_Hypothalamus |
| *WDR60* | rs10081191 | 0.22 | 0.06 | Brain_Nucleus_accumbens_basal_ganglia |
| *WDR60* | rs10081191 | 0.33 | 0.07 | Brain_Putamen_basal_ganglia |
| *WDR60* | rs10081191 | 0.80 | 0.02 | Brain_Spinal_cord_cervical_c-1 |
| *WDR60* | rs10081191 | 0.40 | 0.08 | Brain_Substantia_nigra |
| NES, normalized effect size; SNP, single nucleotide polymorphism. *P* value was generated using a linear regression model between the genotypes and expression levels, and *P* value less than 0.05 was considered to be statistically significant. | | | | |

**Supplementary Table 10** The predicted functional relevance of rs35075890, rs10081191 and the other SNPs in strong LD with them.

(A) The predicted functional relevance of rs35075890 and 6 other SNPs in strong LD with rs35075890 in the present study.

| Chr. | Positions^a^ | LD (*r*^2^) | SNPs | Ref | Alt | *P* values in GWAS | Posterior probabilities | Genes | Functional annotation | Promoter in brain tissues | Enhancer in brain tissues | DNAse in brain tissues |
| --- | --- | --- | --- | --- | --- | --- | --- | --- | --- | --- | --- | --- |
| 7 | 70171159 | 1 | rs3923606 | A | G | 1.77E-04 | 0.22 | *AUTS2* | Intronic | No | Yes | Yes |
| 7 | 70172412 | 1 | rs3936228 | A | G | 7.02E-05 | 0.58 | *AUTS2* | Intronic | No | Yes | No |
| 7 | 70178230 | 1 | **rs35075890** | A | G | 7.02E-05 | 0.36 | *AUTS2* | Intronic | No | Yes | No |
| 7 | 70206035 | 0.49 | rs58967237 | G | A | 0.06 | 0.21 | *AUTS2* | Intronic | No | No | No |
| 7 | 70209300 | 0.81 | rs79299033 | G | A | 1.86E-04 | 0.69 | *AUTS2* | Intronic | No | Yes | Yes |
| 7 | 70211000 | 0.81 | rs6967670 | A | G | 4.09E-04 | 0.36 | *AUTS2* | Intronic | No | No | No |

(B) The predicted functional relevance of rs10081191 and 14 SNPs in strong LD with rs10081191 in the present study.

| Chr. | Positions^a^ | LD (*r*^2^) | SNPs | Ref | Alt | *P* values in GWAS | Posterior probabilities | Genes | Functional annotation | Promoter in brain tissues | Enhancer in brain tissues | DNAse in brain tissues |
| --- | --- | --- | --- | --- | --- | --- | --- | --- | --- | --- | --- | --- |
| 7 | 158299987 | 0.54 | rs10260103 | A | C | 8.45E-04 | 0.00 | *PTPRN2* | Intronic | No | No | No |
| 7 | 158316235 | 0.57 | rs73729669 | A | G | 4.88E-05 | 0.00 | *PTPRN2* | Intronic | No | No | No |
| 7 | 158318988 | 0.93 | rs10949731 | A | G | 4.77E-05 | 0.00 | *PTPRN2* | Intronic | No | No | No |
| 7 | 158319597 | 0.96 | rs7808858 | G | T | 1.49E-05 | 0.01 | *PTPRN2* | Intronic | No | No | No |
| 7 | 158322716 | 1 | rs10081235 | G | A | 8.74E-06 | 0.19 | *PTPRN2* | Intronic | No | No | No |
| 7 | 158322806 | 1 | **rs10081191** | A | C | 8.74E-06 | 0.19 | *PTPRN2* | Intronic | No | No | No |
| 7 | 158325503 | 0.7 | rs4909237 | C | T | 6.29E-05 | 1.00 | *-* | - | No | Yes | Yes |
| 7 | 158325572 | 1 | rs4909238 | T | C | 9.51E-06 | 0.09 | *PTPRN2* | Intronic | No | Yes | No |
| 7 | 158326087 | 1 | rs4909239 | A | G | 9.51E-06 | 0.16 | *PTPRN2* | Intronic | No | Yes | No |
| 7 | 58335121 | 0.98 | rs2335161 | G | A | 1.05E-05 | 0.12 | *PTPRN2* | Intronic | No | Yes | No |
| 7 | 158336474 | 0.98 | rs10266729 | T | C | 9.73E-06 | 0.14 | *PTPRN2* | Intronic | No | No | No |
| 7 | 158337463 | 0.4 | rs10270958 | T | C | 0.08 | 1.00 | *PTPRN2* | Intronic | No | No | No |
| 7 | 158340456 | 0.93 | rs10156201 | A | G | 8.84E-06 | 0.11 | *PTPRN2* | Intronic | No | No | No |
| 7 | 158384590 | 0.77 | rs10277152 | T | C | 1.45E-04 | 0.00 | *-* | - | No | No | No |
| Alt, alternative; Chr., chromosome; LD, linkage disequilibrium; NA, not available; Ref, reference; SNP, single nucleotide polymorphism. Posterior probabilities were calculated by Probabilistic Annotation INTegratOR (PAINTOR), *r*^2^ was calculated between SNPs and rs35075890 or rs10081191 based on genotype data of the Asian population of the 1,000 Genomes Project. Only the SNPs with *r*^2^ > 0.4 were retained. ^a^Positions based on NCBI Build 37. The *r*^2^ was calculated using the HaploReg software (version 4.1). | | | | | | | | | | | | |

**Supplementary Table 11 The allele and genotype frequencies of rs35075890 and rs10081191 in different populations.**

| (A) The allele and genotype frequencies of rs35075890 in different populations. | | | | | | | | |
| --- | --- | --- | --- | --- | --- | --- | --- | --- |
| Populations | Sample size^a^, n | Frequencies of G allele | Alleles, n | | Genotypes, n | | | *P* |
|  |  |  | G | A | GG | GA | AA |  |
| In the present study |  |  |  |  |  |  |  |  |
| Discovery stage samples | 294 | 0.0600 | 35 | 553 | 1 | 22 | 260 |  |
| Replication stage samples | 405 | 0.0670 | 54 | 756 | 1 | 52 | 352 |  |
| Overall | 699 | 0.0640 | 89 | 1,309 | 2 | 74 | 612 |  |
| In 1,000 Genomes Project^b^ |  |  |  |  |  |  |  |  |
| CHB and CHS | 208 | 0.0630 | 26 | 390 | 1 | 24 | 183 | 0.78 |
| African descent | 661 | 0.154 | 204 | 1,118 | 9 | 186 | 466 | 1.72E-09 |
| American descent | 347 | 0.210 | 146 | 548 | 22 | 102 | 223 | 2.79E-16 |
| East Asian descent | 504 | 0.0780 | 79 | 929 | 5 | 69 | 430 | 0.34 |
| European descent | 503 | 0.313 | 315 | 691 | 47 | 221 | 235 | 1.71E-38 |
| (B) The allele and genotype frequencies of rs10081191 in different populations. | | | | | | | | |
| Populations | Sample size^a^, n | Frequencies of A allele | Alleles, n | | Genotypes, n | | | *P* |
|  |  |  | A | C | AA | AC | CC |  |
| In the present study |  |  |  |  |  |  |  |  |
| Discovery stage samples | 278 | 0.315 | 175 | 381 | 26 | 123 | 129 |  |
| Replication stage samples | 410 | 0.316 | 259 | 561 | 49 | 161 | 200 |  |
| Overall | 688 | 0.315 | 434 | 942 | 75 | 284 | 329 |  |
| In 1,000 Genomes Project^b^ |  |  |  |  |  |  |  |  |
| CHB and CHS | 208 | 0.279 | 116 | 300 | 22 | 72 | 114 | 0.18 |
| African descent | 661 | 0.381 | 504 | 818 | 87 | 330 | 244 | 2.00E-03 |
| American descent | 347 | 0.465 | 323 | 371 | 83 | 157 | 107 | 2.51E-09 |
| East Asian descent | 504 | 0.315 | 318 | 690 | 56 | 206 | 242 | 0.99 |
| European descent | 503 | 0.494 | 497 | 509 | 126 | 245 | 132 | 1.48E-14 |
| ^a^Sample size was the number of successfully genotyped individuals. ^b^The 1,000 Genomes Project was based on the release in March 2019. African descent includes Americans of African Ancestry in SW USA (ASW), African Caribbeans in Barbados (ACB), Esan in Nigeria (ESN), Gambian in Western Divisions in the Gambia (GWD), Luhya in Webuye, Kenya (LWK), Mende in Sierra Leone (MSL), Yoruba in Ibadan, Nigeria (YRI). American descent includes Colombians from Medellin, Colombia (CLM), Mexican Ancestry from Los Angeles USA (MXL), Peruvians from Lima, Peru (PEL), Puerto Ricans from Puerto Rico (PUR). East Asian descent includes Chinese Dai in Xishuangbanna, China (CDX), Han Chinese in Beijing, China (CHB), Han Chinese South (CHS) and Japanese in Toyko, Japan (JPT), Kinh in Ho Chi Minh City, Vietnam (KHV). European descent includes British in England and Scotland (GBR), Utah Residents (CEPH) with Northern and Western European Ancestry (CEU), Finnish in Finland (FIN), Iberian Population in Spain (IBS), and Toscani in Italia (TSI). | | | | | | | | |

**Supplementary references**

1. **de Leeuw CA, Mooij JM, Heskes T, Posthuma D**. MAGMA: generalized gene-set analysis of GWAS data. *PLoS Comput Biol.* 2015; 11: e1004219.

2. **Jannot AS, Ehret G, Perneger T**. P < 5 x 10(-8) has emerged as a standard of statistical significance for genome-wide association studies. *J Clin Epidemiol.* 2015; 68: 460-465.

3. **Kichaev G, Yang WY, Lindstrom S *et al.*** Integrating functional data to prioritize causal variants in statistical fine-mapping studies. *PLoS Genet.* 2014; 10: e1004722.

4. **Stankovic K, Rio C, Xia A *et al.*** Survival of adult spiral ganglion neurons requires erbB receptor signaling in the inner ear. *J Neurosci.* 2004; 24: 8651-8661.

5. **Chai R, Li GL, Wang J, Zou J**. Hearing Loss: Reestablish the Neural Plasticity in Regenerated Spiral Ganglion Neurons and Sensory Hair Cells. *Neural Plast.* 2017; 2017: 1807581.

6. **Xia MY, Zhao XY, Huang QL *et al.*** Activation of Wnt/beta-catenin signaling by lithium chloride attenuates d-galactose-induced neurodegeneration in the auditory cortex of a rat model of aging. *FEBS Open Bio.* 2017; 7: 759-776.

7. **Lee N, Park J, Bae YC *et al.*** Time-Lapse Live-Cell Imaging Reveals Dual Function of Oseg4, Drosophila WDR35, in Ciliary Protein Trafficking. *Mol Cells.* 2018; 41: 676-683.

8. **Zhang Y, Liu H, Li W *et al.*** Intraflagellar transporter protein (IFT27), an IFT25 binding partner, is essential for male fertility and spermiogenesis in mice. *Dev Biol.* 2017; 432: 125-139.

9. **Chen Y, Lu X, Guo L *et al.*** Hedgehog Signaling Promotes the Proliferation and Subsequent Hair Cell Formation of Progenitor Cells in the Neonatal Mouse Cochlea. *Front Mol Neurosci.* 2017; 10: 426.
